# Regulating Alternative Healing in France, And the Problem of ‘Non-Medicine’

**DOI:** 10.1093/medlaw/fwy024

**Published:** 2018-06-27

**Authors:** Emilie Cloatre

**Affiliations:** Professor of Law, Co-Director of Research, Kent Law School, Eliot Building, University of Kent, Canterbury CT27NS, UK

**Keywords:** CAM, alternative healing in France, Law, science and medicine

## Abstract

This article explores the ambiguities of the legal system that, in France, regulates ‘alternative healing’, and determines the boundaries of legitimate medical care. While the law suggests that the delivery of therapeutic care should be the monopoly of biomedically-trained professionals, alternative healers operate very widely, and very openly, in France. They practice, however, on the verge of (il)legality, often organising their activities, individually and collectively, so as to limit the likelihood of state intervention. This creates a high degree of precarity for both practitioners and, crucially, for patients. Efforts to change the system are being deployed, but while healers themselves have increasingly organised to seek recognition by the state, alternative healing occupies an uncertain policy space: they are not fully constituted as a social and policy matter by the state, and occupy a liminal position between medicine and spirituality that “unsettles” republican ideals of scientific rationality, and of secularism. This article explores some of those tensions, at the crossroad between law, science, and medicine. It reflects on why tensions seem to persist around the regulatory questions at stake, and suggests that ways forward may depend on moving away from science as a sole arbiter in drawing boundaries of legitimate and illegitimate care in regulation.

## I. INTRODUCTION

This article interrogates the role of law in creating the boundary between legitimate and illegitimate practices in healthcare, and between the therapeutic and the non-therapeutic. It uses the case of the ambivalent position of complementary and alternative therapies in France to ask what happens when practices that patients turn to for treatment do not rest on medical paradigms, and are considered, as a result, as illegitimate by the state. In France, this has at least two notable effects. One is for those practices to fall within a grey area of il/legality, where they are formally legal only when delivered by biomedically-qualified professionals, and otherwise simply co-exist, more or less precariously, with the formal, regulated, healthcare system. The other is to occupy an uncertain policy space: where practices fall outside of medicine, they also seem to stop being a policy matter for most public health institutions. This means that questions of legal ambivalence and precarity cannot be addressed effectively. Meanwhile, legitimacy becomes constructed and negotiated aside (and in spite of) the law, and independently of state intervention.

Throughout this article, legitimacy is understood as always being ‘in the making’, and co-produced by both legal and scientific regimes.^1^ Indeed, a feature of the French system is the persisting tensions between conflicting understandings of the role that science should play in defining the boundaries of legitimate care, and the boundaries between legal and illegal healing. At least in its predominant official settings, the French state continues to rely intensely on scientific paradigms to draw those boundaries, and to resist any move away from such rationale. At the same time, everyday practices are challenging this model, and press for a new form of recognition in law of *difference* in epistemologies of care. Patients, in France as elsewhere, have keenly embraced alternative healing, yet the French state has been slower than others at findings new ways to organise this changing landscape of care and the unsettlement of the ‘golden age’ of biomedicine.^2^ The situation, described almost 20 years ago by Ramsey as **‘***France cling[ing] to what many think of as its Napoleonic Heritage while eagerly embracing medical pluralism*’^3^ offers useful insights into two sets of questions relevant to medical law, and to broader legal scholarship: first, how do practices of healthcare operate on the verge of (il)legality; and secondly how do legal and scientific rationalities work together to define the boundaries of medical care. These questions are important both because of the contemporary urgency of addressing underexplored regulatory tensions around alternative therapies, and because of the rich stories this field has to tell about the shifting boundaries of law and legitimacy in everyday health practice.

The article explores these questions by looking at how non-biomedical healing systems are regulated in France, and how they operate in practice. In the UK context that readers may be more familiar with, those practices would broadly fall under the label of Complementary and Alternative Medicines (CAM).^4^ Such labels however do not bear the same recognition in France,^5^ and indeed terminology has been both sensitive, and illustrative of broader tensions in the definition of medicine and its boundaries: the state has explicitly rejected the label of ‘complementary’ (as biomedicine, it argues, does not need complementing), the label of ‘alternative’ (as an alternative to scientific rationale is deemed dangerous to the health system), or indeed of ‘medicines’, as ‘medicine’, it argues, is only biomedicine (and indeed the term ‘*médecine*’ is often used where ‘biomedicine’ would be preferred in English). The non-medical identity of those therapies (although some official documents note that even calling them ‘therapies’ would be accepting that they have therapeutic value, which would also contravene dominant state narratives) makes them difficult to define, and in turn to challenge. Formally, CAM in France are referred to as ‘*Pratiques non-conventionnelles à visée thérapeutique*’ (‘unconventional practices with a therapeutic aim’). In this article, I reverse to more common labels in the English language, including ‘alternative therapies’ or ‘non-biomedical systems of care’.

Though focused on practices that are precisely defined by law and science as being non-medical, this article aims to contribute to scholarship on medical law. Indeed, at its core is the question of the boundaries of the ‘medical’ in healthcare, the role of law in creating these boundaries, and implications for regulating systems of care that are positioned outside of biomedicine. This has conceptual and policy significance, and may also open reflections on medical law as a discipline, and its borders. As medical law has spent significant efforts engaging with new scientific developments and medical advances, it seems relevant to consider how legal systems can be challenged precisely by the drive away from the biomedical, and its teleological understanding of modern healthcare. At the core of the discussion is the question of epistemological tensions in the laws that surround medicine, and determine its boundaries.

After a brief summary of research methods, the article starts by reviewing the social, policy, and regulatory dilemmas raised by non-biomedical healing in France. It sets those against the current legal framework in France, then moves to comparing this framework with everyday practices, emphasising the significant gaps between what the letter of the law suggests, and its everyday practice, and questioning throughout the meanings of this ambivalence. Finally, it reflects on why tensions seem to persist around the regulatory questions at stake, and suggests that ways forward may depend on moving away from science as a sole arbiter in drawing the regulatory boundaries of legitimate and illegitimate care. In an area that has become so controversial and polarised in France, it should be made clear from the outset that the article does not seek to make normative claims about the role that non-biomedical healing practices should play in healthcare delivery: instead, it positions itself principally as a critical description of an ongoing field of controversy, of which legal ambivalence has become a dominant and problematic feature. From there, it advances some suggestions on how regulatory strategies could be reimagined to integrate better such critical reflections, and to reframe the issue as a social scientific, and socio-legal one, to be addressed through a multidisciplinary approach.

## II. METHODS

The research presented in this article is part of a larger project investigating the relationship between law and traditional and alternative medicines in Europe and Africa.^6^ The article is based on initial findings from one of the project’s case studies, France, compiled from literature reviews; an analysis of the main policy and legal documents available on the topic, including those from public authorities and from the main healing associations; case-law; parliamentary debates; and preliminary interviews with 15 participants, representing a mixture of public authorities and representatives of healing associations (who were all also practicing healers). Ethical approval was granted by the University of Kent Ethics Committee on 1 March 2017. Interviews were open-ended, and aimed to encourage informants to talk about legal issues while placing them in the context of their day-to-day practice. Informants were approached following an initial mapping out of the key institutions involved in regulating the field, representing healing professions or having contributed to specific debates on the topic, and were contacted via email. As the field is highly sensitive in France (in particular due to the legal situation described below), I chose to not record those initial interviews (with two exceptions where informants were particularly open), and privileged instead detailed note-taking. This enabled me to also use those pilot interviews to understand better the sensitivities and stakes of the field. In methodological terms, the aspects of the research presented draw on, and aim to contribute, to two main fields of socio-legal research: first, work that has focused on exploring the interactions between law and scientific knowledge;^7^ second, explorations of everyday practices of illegality.^8^ Those fields are here brought into conversation with the questions of the constitution of the boundaries of legitimate medical care through law and science.

## III. NON-BIOMEDICAL HEALING IN FRANCE AND ITS SOCIAL CONTEXT

In recent years, alternative therapies have become an increasing matter of interest for scholars across law and social sciences.^9^ This is in part due to their continued popularity around the world, with a majority of patients using practices other than those of biomedicine for some of their ailments, but also because of the socio-regulatory challenges they raise. Indeed, they create highly complex tensions between, on the one hand, safety concerns, and on the other pressure to respect patients’ freedom of choice. Patients’ turn to alternative healing is often shaped by cultural traditions, personal experiences, and political wariness of biomedicine as enterprise, which makes decisions in the field highly sensitive. Debates in the field are also always animated by tensions around the role that science should play in medical care: advocates of scientific knowledge as the main or exclusive resource to draw the boundaries of healthcare often clash with those who consider that scientific knowledge should not be the sole arbiter of what constitutes legitimate care. In the UK, and although debates in the field are still far from settled, some of those issues have been approached through what Ayo Wahlberg refers to as ‘differentiation within’, a triage within CAM professions, either through self or statutory regulation, between practices and practitioners deemed legitimate or not.^10^ As he points out, this approach is not unproblematic, and tensions continue to arise around such legitimacies (for example with recent debates around the provision of homeopathy on the NHS).

France, however, provides an interesting contrast with this strategy, and an opportunity to explore more closely both the question of illegality at the borders of healthcare, and of the role of science in co-constituting legitimacy in law and medicine. There, like elsewhere, patients commonly turn to non-biomedical therapies.^11^ Some of the reasons for this include, sometimes alongside each other: a frustration with the biomedical system; an attempt to find more individualised forms of care; a desire to return to a more ‘natural’ approach to health,^12^ or more traditional forms of knowledge; a political drive away from “Big Pharma” and the capitalist aspects of biomedicine; and the revitalisation of popular, local or imported, traditions in healthcare that had been pushed away over the years by biomedicine.^13^ This demand has resulted in what some see as a proliferation of alternative healers—although in the light of the long history of popular medicine in France, it is debatable whether this proliferation is really that, or if it is also a renewed visibility of an alternative healthcare system that has always existed in some form. Nonetheless, social scientists tend to agree that there has been a renewed interest in alternative practices since the post-1968 period.^14^

This increase use, or increased visibility, of alternative healing since the 1970s, has created intense debates and tensions. While some of those were initiated as part of a policy conversation,^15^ in more recent years conversations have materialised as occasional moments of friction between a top-down, disciplinary, emphasis on the need to weave out any healing practice that was not ‘scientifically proven’, and growing lobbying (mostly by healing professionals) in favour of a more open and pluralist system of care. The context of those debates is partly made peculiar in France by the traditionally heavily political role played by the medical establishment. Historians have traced this position to the revolution and the historical alliance between scientific/medical and state rationales, in their mutual desire to break away from the Church.^16^ A particular class of ‘elite doctors’ have, since then, often occupied influential roles in public offices, that have in part shaped official narratives of what constitutes valid healthcare and the role of science within it.^17^ As an example of the occasional moments of tension that arise around alternative healing in France, one of the most recent official conversations took place in a special Senate Commission in 2013.^18^ Although the importance of this particular Commission should not be overplayed, it represents one of very few ‘official moments’ in which extensive conversations on alternative healing took place in France in recent years, and therefore an unusual opportunity to see formal discourses unfold. Transcripts from the lengthy debates and extensive interviews on which this investigation was set give a sense of the tensions surrounding the field in French politics. As an example, one of the interviewees explains:

‘The authority of the healer goes hand in hand with their legitimacy. From the XVIIth century, under the influence of the Enlightenment, the scientific paradigm provided the foundations of modern medicine. Progressively, this scientific dimension removed medicine from the domain of intuition, magic, home remedies and religious beliefs, according to which illness could represent at times the work of the devil, at others redemptive suffering. Do not be mistaken: these misleading therapies that you see today operating as folk, orientalist or other practices, bring us back to this ancestral paradigm!’^19^

While this particular informant was well-received by the Commission, the same cannot be said of those who adopted a less critical stance towards alternative healing, including those suggesting that some research should, maybe, be undertaken to see the contributions that some of those therapies could make to patient care. Debates in the Commission have other noteworthy features. For example, the appeal to the significance of the ‘scientific revolution’ and the need to ensure we don’t abandon its teachings, such as those quoted above, appear multiple times. Discussions around the notion of therapeutic freedom are also of interest: therapeutic freedom is upheld as important, but understood as being a type of freedom that should be exercised only within what constitutes recognised therapies (here, biomedicine). Finally, the way in which non-Western medicines are dismissed throughout remind us of why the question of rationality in medicine is also loaded with cultural, including (post)colonial significance. In reading those debates, it is noteworthy that the person leading the Senate commission was himself a doctor, as well as a senator.

## IV. LEGAL FRAMEWORK: DRAWING THE LINES OF IL/LEGALITY IN HEALING

This section turns to exploring the legal framework that surrounds alternative healing in France, and in which current debates are taking place, and draws attention to three defining features: first, that in principle, only medical doctors or authorised professions (such as midwives, dentists, and nurses, all within particular limits) can practice healing; second, that in principle only pharmacists can sell products that are considered as ‘medicinal’; and third that all other healing professions may be under the surveillance of the Miviludes (*Mission Interministerielle de Vigilance et de Lutte Contre les Dérives Séctaires*), an agency charged with monitoring sects and cults. These three features create a limited system for alternative therapies that, where permitted, are subordinated to biomedicine and restricted to the biomedical professions.

### A. ‘Exercice illegal de la médecine’ and the Professional Boundaries of Healing

Since 1804, and with various adjustments over the years, the law makes it illegal for anyone who is not a qualified medical practitioner to ‘treat or diagnose’.^20^ Where this is the case, sentencing can be up to 2 years imprisonment and/or a 30.000 euros fine.^21^ The terms ‘treat’ and ‘diagnose’ have been interpreted broadly. For example, claiming that a particular intervention is curative, regardless of its potential efficacy can be constitutive in itself of illegal medical practice. In effect, this means the delivery of most alternative therapies, such as folk herbalism, Traditional Chinese Medicine, naturopathy, or acupuncture are formally illegal where practiced by non-doctors.^22^ France is not unique in its approach to regulation through criminalisation, with comparable systems elsewhere in southern Europe having used such prohibitive techniques,^23^ but it is becoming increasingly unusual in a changing regulatory context, and under increasing local pressures to revisit the scope of the monopoly it offers to doctors in providing healthcare. As I return to below, the application of the law in practice is not as strict or clear-cut as the text may suggest—though potentially no less problematic or threatening from the perspective of healers.

### B. Exercice Illegal de la pharmacie and the Sale of Therapeutic Goods

This legal framework is paralleled and supplemented by a comparable monopoly around the sales of medicinal products. Two sets of regulations are relevant here: those surrounding professions, and those surrounding products. The *exercice illégal de la pharmacie* means that anyone other than a qualified pharmacist, and within the space of a pharmacy, is forbidden from selling medicines.^24^ The definition of what constitutes a medicine is broad, and the lines between what is considered medicinal or not by the law are blurry. Any product that makes health claims constitutes a medicine. Medicinal plants and herbal products have been particularly liminal, and complicated by their regulation as products. To summarise here the conditions of their sale: in principle, only qualified pharmacists can sell medicinal plants in raw form, or medicinal products based on plants, since 1941.^25^ An exception used to exist for qualified herbalists, but the relevant diploma disappeared under a 1941 law (issued by the Vichy government). By way of exceptions, a list of plants that can be sold freely was created in 2008, to now comprise of 148 plants.^26^ Restrictions remain as to the conditions of sale for those plants by anyone who is not a qualified pharmacist: in those cases, and apart from a few exceptions, they cannot be mixed, and they should not either carry a message suggesting they have medicinal properties. Only pharmacists are allowed to provide advice, for example on how a plant should be taken and for what ailments. Anyone who provides advice on those plants without being a qualified pharmacist (including in herbalist stores or health stores, for example), could be guilty of *exercice illégal de la pharmacie*. Similar restrictions apply to herbal products that have been manufactured: ‘food supplements’ can be sold by those who are not pharmacists, but either their labelling or any advice provided could see them requalified as medicine, and in turn their provider as illegally practicing pharmacy.^27^

The illegality that can surround therapeutic practices, professions, and products, or those who claim to be such, or look like them, is obviously significant for the possibility of alternative healing to develop officially. On paper at least, the law only allows biomedical professionals and products to respond to the health needs of the population. Others are not allowed to intervene in this sphere, regardless of patients demands as they could be prosecuted. Below, I return to the contrasts between this strict system on paper, and its practice.

### C. Sects, Healing, and State Surveillance

A third feature of the French system, that has as much to do with France’s approach to religion as it has to do with the question of medicine, is the monitoring by the Miviludes of aspects (and abuses) of alternative medicine. The Miviludes is a particularity of the French regulatory environment, and feeds into broader conversations about secularism and freedom of religion that are partly beyond the scope of this article. Created in the mid-1990s (though under a different name and slightly different structure) its aim is to oversee and control what they refer to as ‘*derives sectaires*’—which is difficult to translate being closest in literal meaning to ‘cultish deviance’ but in colloquial terms to ‘sectarian tendency’ or ‘sectarian drift’.^28^ In terms of its structure, the Miviludes is an interministerial agency (‘*mission interministérielle*’) and is operated by a set of permanent members organised in four sub-teams focusing respectively on monitoring sects in the fields of youth and education; security; work and employment; and health (the latter group being constituted of only two full-time members). The groups are coordinated by a President and General Secretary, and supported by administrative services. The actions of the Miviludes and its general directions are also shaped by conversations across Ministries, through an executive committee with members from each relevant ministry, and a *Conseil d’Orientation* made of public actors nominated by the prime minister’s office (including for example parliamentarians), and of representative of key associations, mostly in relation to the youth and education sector . Although a detailed exploration of the Miviludes is beyond the scope of this article, it is worth noting from this brief description that it operates both as a day-to-day surveillance agency, and as a space for higher level policy decision in which complex questions touching on the relations between state and faith-based practices are negotiated.

The rationale behind the Miviludes’ involvement in relation to alternative healing is at one level straightforward, though peculiar in its effects and framing: the underlying assumption is that health has proven a powerful entry point for cult-leaders seeking to recruit new followers.^29^ The vulnerability of those who may be approached as patients before falling under excessive psychological influence is seen as one factor for this. Historically, sects have often specifically engaged with therapeutic, or pseudo-therapeutic, strategies, and leaders have frequently presented themselves as also having healing powers that would enable their followers to live a more healthy and fulfilled life. Of course, the specific context of secularism in France has also a significant role to play in the emergence of a particular discourse and institutional practices around cults.^30^ While the Miviludes does not see its role as being one of monitoring therapeutic unorthodoxy, or even as having to monitor cases of illegal medical practice per se, they normally seek to investigate and/or intervene when they are faced with a situation where the influence of a healer is considered as impacting on the patients’ own judgment, and of tending towards an undue ‘*emprise*’.^31^

Representatives of the Miviludes are very clear that their role is not one of public health, nor to monitor healers that do not fall within that particular, more narrow and more extreme, form of mental control. However, the fact that they have an official role that involves the monitoring of healers, in what appears otherwise as a policy vacuum as I describe below, positions them as relatively central actors. Often, when questions are raised in Parliament about the possibility of softening existing legislation on medical practice to open the door to alternative therapies, or questions about the formal recognition/regulation of CAMs, official answers simply state that alternative healers’ activities are regulated both by the law on *exercice illégal de la médecine*, and by the oversight of the Miviludes. Even though, in practice, folk herbalists or Chinese healers may have very limited contacts with a Miviludes that is not, fundamentally, interested in them except in cases that also have sectarian characteristics, the symbolic of this discourse is significant.

### D. Some Limited Exceptions, within the Biomedical Frame

Before turning to the ambivalence of the above regulatory system in practice, and to some of its effects, it is worth pointing out the spaces that the law creates, by means of exception, for alternative therapies. Those, however, don’t fundamentally change the biomedical foundations of the system, but create some ambivalence. I turn briefly to these two sets of exceptions: first the emergence of osteopaths and chiropractors as the first alternative professions recognised by the French state. And second, the possibility to practice alternative therapies for biomedically qualified practitioners, and within the spaces of biomedicine, such as pharmacies.

#### 1. Emerging professions: Ostheopathie and Chiropraxie

Since 2002 both ostheopathie and chiropraxie have been under statutory regulation, and recognised as health professions.^32^ If prescribed by a doctor, sessions with ostheopaths or chiropractors are refunded through the social security system and private health insurances (at different rates depending on particular schemes and circumstances). The rationale behind the emergence of these two particular professions is not entirely clear: indeed, there seems to be no obvious reasons as far as either paradigm or evidence are concerned to isolate these two professions in a system that is otherwise closed to non-biomedical practices. But several factors seem to have been influential in this recognition. One reason provided by some informants in this project is that, as far as osteopaths and chiropractors were concerned, the law around illegal medical practice had stopped being effectively enforced by the time the state decided to regulate the professions formally. Secondly, these practices are possibly in less direct conflict with the monopoly of doctors themselves, and more clearly on the terrain of physiotherapists (a profession that has been less powerful than doctors in France, but also always subordinated to them). Third, osteopaths and chiropractors have for long been tightly organised as a profession, offering reassurance to regulators that they were well-placed to maintain professional standards within. Indeed, for example, they also became one of the first CAM professions to be statutorily regulated in the UK.^33^ Finally, it should also be noted that, although the model provided by osteopaths and chiropractors is often seen as a model to follow by other professions seeking recognition, to which I return below, it is not devoid of controversy. For some, professional recognition took place too early, before solid structures were in place to check and control schools and diplomas. This has meant that the number of ostheopaths grew with uneven standards across the profession until recently.

#### 2. Alternatives ‘within’

Secondly, although those who are not biomedically qualified are carefully kept out of the healthcare system by the law, actors and institutions that are recognised by biomedicine are able to embrace, to some degree, alternative epistemologies. This applies to doctors, pharmacists, and, arguably, the pharmaceutical industry. For example, as the current regulation of treatment and diagnosis is based on a boundary between those who are allowed (biomedically qualified professionals) and those who are not, rather than prohibiting particular practices, it is legally possible for doctors (and to some extent other health professions such as dentists, nurses, or midwives) to also offer some form of alternative therapies. It is relatively common, for example, for some doctors to provide acupuncture or homeopathy to patients,^34^ and indeed both practices have a long history within medical circles.^35^ When practiced or prescribed by a doctor, treatment will also be partially refunded by the social security system (and often complemented by private health insurances), even though questions persist in determining the rates of reimbursements of particular acts, and controversies regularly arise over such social coverage. Many universities offer further professional diplomas for doctors who want to undertake new training including in some complementary therapies.^36^ When practicing such alternative therapies, doctors remain under the monitoring of their professional association, and need to ensure that they continue to provide care that is considered sufficient and appropriate by their peers, but the practice of alternative therapies itself is authorised. In the case of established practices such as acupuncture and homeopathy, experienced doctors can be highly popular with patients, and their practice highly profitable. In addition, spaces have been carved within hospitals for complementary therapies.^37^ This is not to be said that such practices are not controversial even within the medical professions. For example, on the 18^th^ March 2018, 124 doctors published an open letter in *Le Figaro*, a widely read (politically conservative) newspaper, in which they called for a stricter approach towards their peers who practice homeopathy and other such ‘*fake medicines*’ (as they are referred to): practices that are ‘neither scientific nor ethical but instead irrational and dangerous’^38^ ‘[*ni scientifiques ni éthiques mais bien irrationnelles et dangereuses]’*. The letter demands a series of changes, including for the *Ordre des Médecins* to take sanctions against doctors who practice such therapies, the end of the social security coverage of such practices, and the end of trainings in such techniques in medical faculties. It is yet to be seen whether such letter will trigger changes beyond the social media debates it has generated. For now, and in spite of such moments of controversy, the ambivalent situation in France could be summarised, ironically, as one in which only doctors (and to some extent other health professions) can legally practice things other than medicine.

Similarly, pharmacists remain able to sell herbal and alternative remedies, or indeed medicinal plants. Most pharmacies in France will also display extensive ranges of homeopathic products, phytotherapy, or indeed (though more rarely) medicinal plants. Those are highly popular, and a vast industry thrives both in homeopathy and plant-based medicinal products—as long as their supply is controlled by pharmacists, and restricted to the pharmacy space.

Here, the legal system enables a particular form of pluralism ‘within’, that creates some ambivalence as far as the state’s approach to alternative therapies is concerned. At one level, of course, and as is often reminded by public authorities, possibilities are created to respond to the demands of patients that seek more, or something else, than biomedicine. But at the same time, the maintenance of such alternatives within biomedicine suggests that they may, in fact, not be fully ‘alternative’. In turn, this seems to underestimate the degree of change that patients looking for alternative medical practices are effectively demanding: for example patients driven to alternative practitioners may do so precisely in order to escape the biomedical institution. Similarly, the logic of expecting that someone who aims to practice within an alternative system of thought and healing should also have been trained in biomedicine is also questionable: on the one hand, the aim here for the state is to ensure that such systems of care are provided by professionals that operate within appropriate standards, with training that would limit the risk of them failing to diagnose properly, and by professionals that are also subject to the oversight of established professional regulatory bodies. From this perspective, opening a door for doctors to practice other therapies makes sense, as does the requirement that those practicing other therapies should be doctors. But on the other hand, it may not seem so logical to expect that someone who wants to practice according to the precepts of Chinese medicine, that are so fundamentally different from those of biomedicine, should have first been trained in the contrasting and maybe incompatible knowledge of biomedicine.^39^ A regulatory logic is also often put forward to justify limiting the possibility of ‘alternatives’ to within the institution: notably, that since doctors are already overseen by the *Ordre des Medecins*, there is a form of regulatory control over their activities, and a lesser risk of problematic practices. While this is an important argument from a regulatory perspective, there is in principle no reason why such professional body could not be developed for other professions. Indeed this has been, for example, the route taken in the UK in regulating alternative professions ‘within’. Similarly, the possibility to access some forms of ‘softer’ or ‘more natural’ treatments within pharmacies responds to some degree to patients’ demands. But, at the same time, it remains a very limited response that misses some key social elements: first, once again, the fact that patients in seeking natural options may very well also be seeking an alternative to the clinical dimension of the biomedical system—and may be drawn towards health stores rather than pharmacies when doing so. Secondly, the fact that, politically, some of the resistance to biomedicine that has fuelled alternative therapies movements has also been a resistance to its industrial dimension. Finally, from an analytical perspective, the subordination of alternative practices to biomedicine also reminds of the classic argument that biomedicine as an institution is a colonising enterprise—as, indeed, is law.^40^ Here what it means is that long-standing imported therapeutic traditions (such as Tibetan or Chinese medicine), or popular traditions that have historically emerged against biomedical elites, become conditioned by biomedicine, and can only be practiced by those who also hold biomedical knowledge.^41^

Overall, a certain paradox appears in the French system, that is fully embedded in the law itself. Within the biomedical sphere, alternative therapies are able to flourish, and are both popular and often, lucrative, for doctors, pharmacists and the pharmaceutical industry. At the same time, apart from the exceptional case of osteopaths and chiropractors, when operating outside of biomedicine, alternative therapies are legally considered as problematic, and indeed, criminal.

## V. NEGOTIATING PRACTICE AT THE BOUNDARY OF THERAPEUTIC LEGALITY

The legal system surrounding alternative medicine in France, and the broader public authorities discourses that sustain it, therefore appear as ambivalent: on the one hand prohibitive and highly wary of alternative healers; at the same time opening spaces for alternative practices to thrive within and under the control of biomedicine, and when operated by its agents. As is often the case, the lived experience of the system is far from aligned with the letter of the law. In practice, alternative therapies are commonly practiced in France by non-doctors, relatively visible, and very popular. It is not clear that the law has the effect of limiting their use—though it certainly affects experiences: due to the letter of the law, these practices operate at the border of il/legality. Some activities are fully illegal, but may be tolerated, while others find ways to negotiate with the law so that they remain, arguably, formally legal. If the effect of the law has not been to limit neither demand nor supply, it has shaped the way in which alternative practices are framed, and the conditions of their use. Through their activities, practitioners have developed legitimacy-making techniques away from those drawn by the states. They have organised, both with a view to avoid legal crises, and in order to work towards a change to both the social positioning of their practice, and, ultimately, the law. At the same time, this system of negotiation is far from satisfactory from the perspectives of healers themselves, public authorities or indeed, patients. This is particularly so as practices operate within a relative policy gap, where instances and modes of policy reflection have been highly limited, and predominantly premised on the centrality of science in resolving ongoing controversies. In this section, I explore some of these issues and what being ‘non-medical’ means in terms of legalities,^42^ before turning to the place of science in those conversations. I argue that, contrary to what policy practice in France has suggested so far, the complex legal issues at stake cannot be resolved through science itself. They reveal deep socio-cultural tensions that may be better addressed through the tools and knowledge of social science.

### A. Using Alternative Therapies in France: From Legal Restrictions to Widespread Use

Despite the legal framework suggesting that only doctors should treat patients, alternative healers are as easy to find in France as elsewhere.^43^ For example, if a patient in France wants to find a naturopath, a Traditional Chinese Medicine practitioner or a *magnétiseur*, they will have no problem locating them online, in the yellow pages (under a general label of ‘other health professions’), or indeed by walking through the streets and noting the signs on buildings and letterboxes.^44^ Many providers will advertise quite proactively their services, in local papers or leaflets left in public places.^45^ Often, and in part due to this visibility, patients will not even be fully aware that their providers may be practicing unlawfully, or on the verge of illegality.

Occasionally, some healers may work with other (biomedically-trained) health professionals. For example, in addition to hospital staff (doctors or nurses) offering some complementary treatments and techniques, some hospitals bring external therapists who are not necessarily health professionals themselves. As an example, the Hôpitaux de Paris have offered sophrology, Shiatsu and elements of Traditional Chinese Medicine to their patients, as a way to explore the possible benefits these might bring—something that the above mentioned Senate commission was incensed by.^46^ Herbalists interviewed in this project also reported working with both local hospitals and GPs, and occasionally offering some training to health practitioners keen to understand better herbal remedies. If such collaboration with biomedical staff is not exceptional, it is of course not either the only way in which biomedical professionals may view alternative therapists: for some, alternative healing continues to be little more than a waste of resources for gullible patients. Finally, alternative healers are self-employed professionals who are expected to pay taxes, can be offered professional insurance, and some private health insurances have started to reimburse some of their consultations. In other words, in spite of the legal precarity of their activities, they are far from discreet, underground actors, which suggests at least a degree of tolerance by the state, and an ambivalence in their socio-legal positioning.

Of course, as elsewhere, the range of therapies and therapists available to patients is also highly varied. Herbalists, naturopaths, Chinese healers, non-doctor acupuncturists, *magnetiseurs*, sophrologists, coexist with smaller movements of emerging therapies. If some seem far away from the most wary descriptions provided by state officials, the figure of the ‘dangerous healer’ that animates the regulatory system is not entirely imagined. Alongside established practices and professions with a shared system of ethics and practice mapped onto those of existing health professions, who are keen to position themselves as only a part of a broader health system, more obscure movements have emerged that make radical claims about the miraculous powers of a particular individual or technique, and drive patients away from the broader healthcare system. Similarly, individuals advertise self-proclaimed abilities without any attempt to refer to training, or professional attachment, and constitute a new form of ‘quackery’ both for other healers and for the state. Such figures create important concerns for any regulatory strategy: they constitute a reminder of the necessity of drawing lines of legitimacy in healthcare; they also create the need for other practitioners to engage and participate in the drawing of those boundaries.

### B. The Limits of Legal Enforcement

Overall, the practice of alternative healing in France is both common and visible, stretching well-beyond the spaces that the law has allocated to it. It is at first sight in clear contrast with what the law seems to suggest, in its assumption that the health system can only be operated by biomedically-trained health professionals. At least two factors are relevant here. One, highlighted in particular by public actors met in the course of this research, is that legal enforcement in the field is difficult. Even though the law seems sweeping in its claim that only doctors can treat or diagnose, and even though the courts have broadened these terms, as we saw, to encompass claims that a particular act has therapeutic value, providing evidence can be highly complex. Often, it will depend on a witnessing of the act that is rare in the context of private consultations. In addition, and as I return to below, practitioners have also learnt to carefully craft their language to avoid being caught in the definitions of the law.

This has meant that, in practice, enforcement tends to focus on practices where such space for negotiation is more limited, and where the nature of the act offered is in itself easier to classify as medical. Acupuncture is a case in point and indeed one of the activities where non-medically qualified therapists have been regularly prosecuted: the inserting of a needle in the skin is, per se, a medical/therapeutic act and its provision by those who are not authorized (doctors, dentists, and, more recently, midwives), illegal.^47^ Elsewhere, case-law suggests that the law has been enforced mostly in cases of deceit and abuse, that went over and above the everyday use of most alternative healing, and often over long periods of time. However, informants also insisted that prosecutions were in practice highly unpredictable, and that there was always a degree of uncertainty about when prosecution may be started (even when, later, charges were dropped). They argued that the likelihood of prosecution would also tend to depend on particular local circumstances, and often come in waves depending on the convictions or concerns of local prosecutors and local representatives of the *Ordre des Médecins*, or on activities within a particular sub-region. This sense of unpredictability added to a feeling of precarity in practice that I return to below.

### C. Negotiating Tolerance

But in addition to the difficulty of enforcement per se, and as is common in such situations of persisting illegality,^48^ healers have also found subtle ways of working around the law. They have learnt, collectively and individually, both through vernacular interpretations of the law and with the help of expert lawyers, to deploy techniques that enable them to remain on the right side of the law. For example, the vocabulary they use to describe their acts is often carefully crafted, to avoid appearing too ‘medical’ or even ‘therapeutic’.^49^ They may write ‘lifestyle advice’ to their patients rather than offer ‘prescriptions’. They may prefer to stay clear of written advice altogether.^50^ Increasingly, healers also organise and share experience and support in coping with the law: over the years, professional organisations have grown and offer professional registers, legal advice, and codes of practice for particular types of healers.^51^ Naturopaths, herbalists, Traditional Chinese healers, have organised in federations, unions, or associations that look after their professional interests, and seek to develop a parallel system of regulation which, while not recognised by the state, provides a shadow regulatory framework in which those professions operate. Training programmes commonly also include a ‘legal’ component. Individually and collectively, those emerging professions have learnt to ensure that their practice can co-exist with the law.^52^ The negotiation of il/legality is a professionalised and collective enterprise.

Techniques of negotiation with and avoidance of the law are further enabled and sustained by external factors that informants tended to present as some of the reasons behind the apparent level of tolerance from the state in practice. One hypothesis that participants commonly presented, is that a range of interests, beyond those of healers, benefit from alternative healing practices, and that this affects the drive for further enforcement, in particular at the local level. For example, a common assertion is that local authorities derive financial benefits from those activities: although the role that this may play in enforcement and/or tolerance (in particular with in mind the multi-layered and complex nature of ‘the state’) is difficult (if not impossible) to establish with any certainty, a substantial economy has developed around alternative healing, with local ramifications. As an example, fairs and conferences on alternative healing are highly-profitable enterprises that inevitably gather multiple sets of financial interests. Aside from such economic stakes, some informants asserted that local authorities may derive benefits from alternative healers in terms of healthcare provision: the lack of doctors has been a constant issue in France in recent years, in particular in rural areas where communities can be left with no medical or healthcare support (les ‘déserts médicaux’). As a result, alternative practitioners are perceived as able to somehow ‘fill a gap’ in local healthcare delivery and local authorities as unwilling to interrupt their activities for this reason. Of course, such assertions are difficult to verify, and this is beyond the scope of this article. But they offer a sense of the many conflicting interests that contribute to the complexity of the questions of enforcement. In addition to these issues, and fundamentally, it should be noted that in spite of moments of friction, alternative healing is simply not always seen as a priority issue either at the central level of the public health system, nor at the level of local decision-making: often, other more pressing issues or unfolding crises are prioritised in the allocation of time and resources. This explains the apparent ambivalence of the legal system but also, maybe more problematically, the broader policy gap that surrounds the issue in contemporary debates. Overall, deliberate tacit acceptance and simple lack of interest for the issue are difficult to disentangle when seeking to explain lack of enforcement, but both are likely to participate in the current situation quietly persisting in its ambiguity.

### D. Problematizing (Il)legalities

The system as it stands has significant limitations, that all informants, though from opposite perspectives, emphasised: de facto a largely unregulated sphere of activity has developed in healthcare, on the fringes of legality, and with little state oversight. However, when defining what issues those difficulties rest on, and maybe unsurprisingly, actors take fundamentally opposite views, in which the role of science is imagined in contradictory ways.

For public authorities, or biomedical or public health actors, this unregulated dimension is highly problematic because it means that it is possible for patients, relatively easily, to consult outside of the biomedical system for their health requirements; when they do so, there is no centrally-shared standards, codes or regulations that can be imposed upon therapists; there isn’t either any formal guidance on how those therapists should relate to the mainstream health delivery system. This means that the expectation by the state that healthcare should be fundamentally organised around scientifically-proven practices is far from being satisfied: in practice, endless possibilities are available for practices that have not been scientifically proven, and for healers deemed illegitimate, to co-exist with the poorly enforced legal system, in the context of everyday healthcare practices.

From the point of view of healers, the situation is also deemed unsatisfactory. The fact that criminal law is rarely enforced as far as illegal medical practice goes is not enough to fully reassure those who constantly need to negotiate with that possibility. Even if rare, prosecutions do happen, and stories are fast-spread. The fact that they are difficult to predict makes things more problematic for those who are seeking to practice. Of course, even if prosecution is rare and if sanctions are often much lower than what the law suggests, as public authorities emphasise, its *possibility* creates a sense of insecurity: legal consciousness scholarship has long reminded us that those who are faced with the law can be scared and intimidated in ways that go beyond what the legal system may see itself as doing.^53^ In the eyes of the law or public authorities, a prosecution that results ‘only’ in a warning, or ‘only’ in a fine, may have been a non-event. For those who are faced with the law, the same event may be much more remarkable. Similarly, being formally considered as an ‘outlaw’ is symbolically problematic for the many healers who consider themselves as good citizens and aim to operate within shared codes of practice and ethics. For healers and their advocates (a small pool of expert-lawyers with clearly aligned sympathies), this precarity and illegality in practice is often presented as being in breach of their, and their patients’ ‘freedoms’ (often in a libertarian understanding).^54^ There, illegality also enables healers to be positioned as victimised figures, which can benefit their visibility and notoriety.^55^ It is possible, in this way that illegality fuels interest and opportunities.

Finally, the situation is problematic from the perspective of patients. A patient seeking an alternative healthcare provider is effectively entering a field in which standards are unclear, and official guidance limited, other than general reminders by the state of the dangers that alternative medicines may present. Since, officially, only doctors can treat, there is no formal recommendation as to what may be more or less dangerous, or indeed more or less beneficial: for the state, the only lines that matter are those between biomedical professionals and others.

To these well-reported difficulties, we could add the question of legitimacy-building in healthcare. Until official conversations on the regulatory issues at stake emerge, the making of legitimacy (albeit fragile) is negotiated aside and away from public health policies, and in turn not necessarily following any coherent strategy that the state would have some control over. In addition, a striking element in the current polarisation of the debates, between public authorities and healers, is the fact that the social complexity of the relationship between knowledges, law, and medicine is left untouched by both sides. Similarly, pragmatic approaches that acknowledge at least that the debate is not necessarily ‘solvable’ (either by an appeal to science as arbiter, or to a disembedded and abstract idea of freedom), but could be mediated, are noticeably absent from public conversations. In turn, important issues are left aside from the policy sphere: for example, in relation to the negative impact that laws that cannot be enforced in practice may have in hiding from view certain practices (therefore making patients more vulnerable) or in relation to the fact that freedom of choice is never a purely autonomous and abstract idea, but is always embedded in everyday life, and conditioned by social (im)possibilities.

The next section turns briefly to some of the strategies deployed for law reform by healers, and to a lesser degree by state entities, before interrogating a key factor in the underlying tensions surrounding the il/legality of alternative medical practices: their positioning in relation to scientific knowledge. In turn, the article concludes by suggesting that a way forward in the current deadlock of ongoing conversations would be for policy-makers to revisit the role that science can play in developing strategies in this field and, indeed, its limitation as a source of societal knowledge.

## VI. REIMAGINING THE LAW: CONFLICTING DEMANDS AND POLICY VACUUM

The limitations of the current system have inevitably led to calls for it to be revisited, though these have pulled in radically opposite directions, generating high polemics.

### A. Opening-up the Law

In response to their frustration with the current legal system, healers have organised to seek legal reform, developing a range of strategies in their claims for recognition. Here, for many, the road to legitimacy is seen as being ultimately dependent on a change in legal position. First, the many guidance and codes of practice that some professions have issued (eg herbalists, naturopaths, Chinese healers) aim not only at information, self-regulation, and legal advice, but also at facilitating a process of legal recognition. Their expectation is that appearing as a carefully self-regulated profession with shared standards is a necessary first step towards recognition by the state, and in turn would make such recognition faster to implement. Effectively, professions have here been working towards what Wahlberg calls ‘differentiation within’, seeking to front-load a process that has elsewhere been associated with such state regulation. In reference to the UK system, Wahlberg therefore reminds us: ‘*As various CAM therapies come to be mainstreamed into national health delivery, their practitioners are increasingly being called upon to help the public distinguish between the competent and the incompetent within a plurality of different forms of medicine*.’^56^

Second, professional organisations regularly lobby and seek potential support in their quest for recognition. Members of Parliament that appear to be open to their claims are enrolled in bringing them to parliament. Healing associations also work in collaboration with relevant networks at international and European level, and associations abroad (and notably in countries where they have received more official backing), to seek further support. Those international networks are seen as particularly important by groups who view the French system as uniquely restrictive—though it is less clear which weight those external arguments may have on the particular rationalities of the French state. Finally, indirect means of lobbying are also in the making at all times: for example through systems of accreditation for courses or schools that are not specifically relevant to the health professions, or through affiliations or endorsements that create a sense of external legitimacy.^57^

Although it is beyond the scope of this article to develop further these law-making techniques, a few features are worth mentioning. First, although there is some degree of coordination between alternative healers’ associations in the process of recognition, claims and lobbying remain quite heavily articulated by individual professions. Different proposals or demands are put forward, for example, by naturopaths, Traditional Chinese healers or herbalists, who create separate alliances—even though the substance of their legal claims overlap to some extent in their demands for a different form of health professions regulation. At the same time, a handful of professional lawyers take part in those conversations and across different professions. They replicate claims and techniques with a reading, as described above, that is heavily framed around what they see as the infringements of the system on individual freedoms, but appear less willing to explicitly engage with some of the core problems of this regulatory field: for example, that of what could constitute reliable knowledge to differentiate between legitimate or illegitimate practitioners, or legitimate and illegitimate claims and practices, or indeed what pragmatic solutions may be deployed to balance public health interests with individual beliefs.^58^

### B. Policy Gaps and the Limits of State Interventions

Next to the high-level of activity and lobbying by healers and their associations, state strategies in the field of alternative medicines seem strikingly limited. Individual moments of friction and polemics do occur, and the state’s apparatus in the field is, on paper at least, of significance. But, in spite of a short-lived wave of interest in the 1980s,^59^ no clear space seems dedicated to approaching the question of alternative healing as a matter of policy concern. In part because of an emphasis on those practices being outside the medical sphere, and kept at a distance from patient’s care, they fall outside the remit of the main public health entities, and in turn seem to fall between areas of competence, with a few exceptions. First, the Miviludes has effectively been charged with providing some of the key guidance and documents relating to alternative healing and ‘*les dérives sectaires’*. However, the institution is both understaffed for the scale that a broad engagement with alternative healing could represent (with two full-time advisers only dedicated to health practices), and very clear that its scope only involves practices that can be classified as ‘cultish’ rather than alternative healing as a whole. Their role is not about public health. Second, a small subpart of the *Direction Générale de la Santé* is in charge of the scientific evaluation of alternative healing techniques: the *Groupe d’Appui Technique sur les pratiques conventionnelles à visée thérapeutique*, ironically a sub-part of the *Bureau de la qualité des pratiques et recherches biomédicales*. I return to how this focus on scientific evaluation fits with the broader question of the regulation of alternative healing below. Finally, in 2013, the *Centre d’Analyses Strategiques* (CAS), a public think-tank formally independent though institutionally connected to the Prime Minister’s office, released a short report on the place of alternative therapies in France. CAS’s role is to submit reports on a variety of issues in current affairs that could warrant some policy attention. They are non-topic specialists, working across a range of issues, and only some of their proposals will ultimately be taken up by the government for further research or policy discussion. In that respect, the report they produced on alternative therapies is one among many, and not the result of any longer-term agenda or policy strategy. Nonetheless, it still stands as a unique document in which a proposal for a reassessment of current strategies, and a cautious step towards a collective reflection, was put forward.^60^ To this date however, the report has not been seized upon by health authorities and is therefore no more than a set of suggestions. It was, however, intensely scrutinised in the above mentioned Senate commission, in a stark reminder of the polemic nature of the debates. There, in spite of the report suggesting little more than a reflection on a policy area where the legal system simply does not map out on societal experiences, the authors were most vehemently criticised by the commission for their ‘dangerous’ proposals.^61^

In the section below, I argue that the relative lack of engagement of the state with the issues at stake is also symptomatic of a reduction of these policy issues to questions of ‘objective scientific knowledge’. While science can provide useful pointers in setting the boundaries of legitimacy in healthcare, it is not sufficient to answer the societal challenges that surround alternative practices. In an area as heavily shaped by socio-cultural and historical influences, it is essential for states to be willing to engage with the difficult question of social legitimacy in healthcare, and to relocate the role that science can play within this—and indeed its possible limitations.

### C. Knowledge and Negotiating with Science

This section turns to the fundamental question of where science ‘sits’ in the regulation of alternative healing in France. It argues that a heavy focus on science as arbiter of regulatory dilemma has resulted in an insufficient policy engagement. Effectively, biomedicine has constituted the main source of knowledge used to draw the boundaries between legitimate and illegitimate care. This reliance on science is particularly marked in the French public discourses, and explains some of its peculiarities. For example, the senate debates demonstrate deep-rooted fears that the Republican ideal of a rational state may be fundamentally threatened by a move away from biomedical professionals as the only providers of care:

‘The XVIIIth and XIXth centuries have seen the transition from ‘l’Hotel Dieu’ to the ‘public hospital’. The legacy of the Enlightenment has enabled the development of Western, scientific, medicine. The XXIst century may see a descent from a the ‘Public Hospital’ to the ‘altar of gurus’. The Lights would then be extinguished by the sectarian obscurantism that imaginary therapeutic methods disseminate to an eager, suggestible, public!’.^62^ ‘Relativism makes superstititions appear as valid as sciences. Science will even be relegated to mere myth, and criteria of rationality will now be presented as one element among many others by a relativist culture. We will be brought right down!’^63^ ‘Since the beginning of the last third of the twentieth century and the experience of totalitarianism, the legitimacy of the scientific basis has been challenged, as if we should forget the need for the “clinical” based on the use of placebo and the so-called “prospective, randomized, double-blind” studies. Soon, postulates will replace evidence.’^64^

Republican ideals are here being replayed in the particular context of healthcare. In turn, state responses to the question of alternative medicine have similarly focused on establishing the scientific validity of alternative therapies, through the tests of biomedicine itself—this is not unusual as such, but what is peculiar in France is the absence of other visible sources of regulatory inspiration. Science is posited as the best or only way to regulate the boundary between legitimate or illegitimate healthcare. If we are to approach the question as not only a scientific one, but also a social one, however, this may be insufficient.

Of course, the question of scientific validity is crucial to health delivery. At the same time, patients’ use of alternative medicines is not only about science, or indeed may be shaped by a social rejection of the clinical gaze. There is a degree to which patients’ demand for alterity cannot be fully answered as a question of science. This plays out at two levels: first, the set of tests and evidence deployed by biomedicine may not be sufficient to engage alternative ways of knowing. Second, where patients’ choices differ from those of the state, or from the offers of science, there may be possibilities to accommodate those choices within public health objectives rather than seek to prohibit them, or fight them off. In other words, even if sceptical of alternative medicines, the question of regulation may need to rely on knowledges other than those of science and biomedicine, including those of the social sciences. So far, the French state has effectively focused on prohibition in law, if not in practice, as its main strategy for dealing with those that fall outside of the scope of scientific proof. In turn, this has meant that vast areas of therapeutic practice have been left entirely free from state regulation or intervention. Similarly, it has meant that there is no sense of hierarchy in the eyes of the state between different non-biomedical practices, or different professions and no official position as to what may be more useful to particular conditions, or what, indeed, may be more dangerous or unreliable. In the eyes of the state the only relevant line to determine officially the borders of legitimacy is between what is considered as ‘scientific’ or ‘unscientific’, and who has been sufficiently trained in biomedicine to practice healthcare.

Alternative healers themselves have placed science at the centre of much of their narratives, though their relationship with scientific knowledge is ambivalent. On the one hand, they emphasise the shortcomings of biomedicine, and position themselves as offering something different, resting on different understandings of the world. On the other hand, in order to enter the predominant medical system, they are keen to demonstrate, in this system’s own terms, the validity of their claims. Scientific language is deployed to that effect, and an effort to try to develop forms of scientific evidence in order to back their claims is central to a vast part of the legitimation strategy.^65^ Inevitably, such balancing within professions is done in a myriad of ways, with sub-groups of healers focusing more heavily on either difference and tradition, or scientificity.^66^ At stake is the careful balancing of credibility in the eyes of a potential regulator, and the abandonment of difference, that has similarly played out in other contexts. For example Iyioha reminds us that: ‘*The statutory recognition of osteopathy and chiropractic in Britain mandated the rejection of the esoteric foundations of these therapies*’. When negotiating scientific and alternative identities, healers need to decide how far they are willing to be transformed in order to be regulated, and how to negotiate adjustments and identities.

A difficulty in the current framing of the issues at stake, in particular by the state, as scientific, and on their answers depending on ‘scientific evidence’, is that it fails to engage with the social issues that drive patients towards alternative epistemologies of care. In turn, establishing how different techniques can be regulated or deployed alongside those proven by science requires a different set of skills or knowledges, including those that social scientists and anthropologists who have engaged with the question of non-biomedical therapies have developed over the years. This in turn requires that the question be elevated in adequate policy arenas, and revisited as a social problem in need of urgent engagement by the state, across knowledges and disciplines. At present, it appears that the French state’s main responses, in a context of fear that engaging beyond science is a threat to science as an institution, has been to rely on biomedicine to continue to set its own boundaries of acceptability. In turn, this has meant that the space in which proposals for change could be drawn or reimagined has been left to more vehement advocates of alternative healing. A more pragmatic approach, and more consistent with the state’s desire to maintain non-partisan knowledge at the core of its strategy, would be to facilitate a pragmatic and cross-disciplinary engagement not only with scientific knowledge itself, but also with social scientific knowledge well-placed to approach the question of diversity in healthcare as a social phenomenon.

## VII. CONCLUSION

The current legal framework that surrounds alternative therapies in France is riddled with ambivalence. It is constituted of a formally strict boundary between the possibilities for bio-medically trained professionals to offer a broad range of therapies (including some complementary and alternative practices), and a prohibition for most others to treat or diagnose patients—arguably the core elements of healthcare practice. The lived reality of care, however, contrasts with this legal framework: alongside the legally accepted practices of (for example) doctor acupuncturists or homeopaths, patients visit vast numbers of practitioners who do not fit the boundaries set by the law. When they do so, and as they are formally outside a legal system that operates as a blanket prohibition, there is little guidance on what may be more or less reliable, or more or less dangerous, practices or providers. In the eyes of the state, those that are not sufficiently proven through science are unable to provide the form of healthcare that it seeks to promote.

Healers have learnt to negotiate this illegality, and over the years developed both individual and collective tools to maintain the ambivalent consensus in which practice can remain possible, and widespread, in spite of its formal illegality. At the same time, they remain unsatisfied with a system that rests on a degree of precarity in practice. Others, who are concerned about the potential risks for patients of this unregulated sphere of care, outside of the biomedical system, are equally frustrated by the system, and emphasise the need for better enforcement. As it stands however, no clear policy space has been defined to explore possibilities for regulatory solutions, maybe precisely because of an insistence that alternative practices cannot constitute a resource for healthcare. Meanwhile exchanges continue to be polemical, between two sets of highly polarised positions either defending an abstract idea of ‘freedom of choice’, or the centrality of science in the provision of care, and the dangers that any dent to that centrality could represent. There has not yet been, in France, a pragmatic engagement with the regulatory crisis that surrounds alternative healthcare. Such engagement is well-needed, but also requires resources that science alone cannot give.^67^

Addressing the crisis of law’s approach to the boundaries of medical care is complicated in part because it requires a reimagining of healthcare as social space, and in turn of the relationship between law and medicine as one that needs to be defined with tools other than those of science. Here, the challenge to law is not a medical one, nor a scientific one, and its resolution is neither determined by science, nor a necessary threat to the scientific consensus. Instead, the question is on how to accommodate practices that are not proven through science itself, but that patients do, nonetheless, choose to use, and how to design systems of regulation that acknowledges this situation without necessarily displacing the importance of biomedical knowledge at the core of healthcare delivery. At the core of those debates, fundamentally, are questions about how the modernity of medicine can be imagined in law: competing visions between those who seek to maintain a vision of modernity as scientifically driven, and those who seek to accommodate alternative understandings of modernity as multidirectional. Here, the challenge of the contemporary state is to reflect on how to accommodate understandings of modernity of which returns to traditions, nature, and movements away from science are an important part. For laws that surround medicine, the challenge is to explore possibilities to produce a regulatory system that acknowledges that societal tensions are resisting the teleological assumptions of science. While many may object with the challenges to scientific knowledge that are driving these, learning to negotiate with those challenges and to draw new lines of conditioned legitimacy may prove inevitable.^68^ A range of pragmatic, non-partisan options can be designed, but they require an engagement with both social scientific knowledge on alternative healing practices, and their socio-cultural complexities, and a clear policy space in which those can be explored.

It is of course not possible in the space of this article to develop definite proposals as to the shape that such engagement should take, and there is no single silver bullet strategy. A few suggestions, however, can be made, that I will articulate here in four key points. First, as suggested above, engaging the issue of the regulation of alternative medicines requires a cross-disciplinary conversation. It is essential that a range of expertise be drawn upon in order to move the debate away from a reduction to questions of scientific evidence only. Ideally, this would be coordinated by public health authorities (for example under the auspices of the Ministry of Health), but also involve researchers with the knowledge required to engage both the question of regulation in its complexity and of the societal dimension of health. Second, the task of such space should in the first instance be one of ‘opening the box of knowledge’ on the matter: as this initial paper has sought to demonstrate, the current ramifications of the legal system in its practice are highly complex, and much remains to be learnt (including, in the remainder of the particular project on which this paper is based). However, from what we know already of alternative therapies, and indeed from some of the intense debates that emerge occasionally in France, it is clear that the problem is a societal one, and one of public health, that should be framed and taken seriously as such. As cross-disciplinary conversations on the matter are taken forward, it is crucial to thoroughly use social sciences research in the field, or indeed commission new research, and bring them into conversation with public health authorities. The creation of the *Groupe d’Appui Technique sur les pratiques non-Conventionnelles à visée therapeutique* previously mentioned was a first step in the direction of commissioning new research in this area, but the answers sought are limited to questions of scientific proof and efficacy, which as suggested here is only one aspect of the issue at stake. This could be paralleled in the social sciences, or where research already exists, it could be brought into conversation. Third, a specific element of attention and conversation should be, precisely, the question of evidence. As argued here and by others, scientific evidence and proof of efficacy are not always sufficient to solve questions of legitimacy, or even usefulness, in healthcare. Patient-centred strategies relying, for example, on qualitative methods and patients’ experience rest on a different set of assumptions about effectiveness (rather than efficacy only). The question of the placebo effect could also be re-opened as one that is not only a sign that something should be dismissed as ‘unscientific’, but as a particular form of social effectiveness. This does not preclude further decisions about what to subsidise or not under the social security system, but could enable a different way of thinking about the effects the healthcare system (as being about curing but also about other forms of caring and wellbeing). It does not either suggest that different systems of proof inevitably need to be seen as being of equal value (and therefore that we should, as suggested in some of the previous quotes, abandon ‘scientific rationality’). Instead, it can mean that different uses could be found for different forms of complementary knowledge. Fourth, and finally, the question of legal and regulatory techniques needs to be given careful attention in these conversations. Fundamentally, the issues explored here are about how legal systems can be reimagined to provide a sustainable system of regulation, that accounts for the social context of practice. Socio-legal knowledge is essential here, and needs to be carefully brought into the debates. One of the challenges of any opening-up of the system is going to be to decide where and how ‘red-lines’ should be drawn. Indeed, relying primarily on scientific methods and the testing of efficacy means that it is relatively easy to draw lines of acceptability, or to define what comes to be considered as ‘real’ or ‘fake’. If we are to open-up the legal system surrounding medicine to new forms of evidence, and expand the role of sociological knowledge and qualitative methods in regulatory strategies, an effect will inevitably be to complicate the drawing of the boundaries of legitimacy. The regulatory system would need to define more nuanced lines to determine who or what can have a role to play in a broadened understanding of health and care, and under which conditions. It would need to reimagine such issues as how criteria of legitimacy for emerging professions could be defined, and how those would map onto institutional formations; how to determine the scope of il/legitimate claims if we are to move beyond a narrow focus on scientific efficacy; who should be allowed to use particular titles or claim particular competences; purpose-built systems for monitoring and enforcement (and how to define the respective roles of the professions and the state); new types of regulation on advertising and patient information etc. These questions invite careful attention to regulatory techniques, moving beyond a reliance on a legal/illegal boundary that appears to be difficult to sustain and to be creating everyday uncertainty. The project on which this article is based will continue to explore such issues, both in France and in each of its other case studies, seeking to put into conversation the ways in which comparable dilemmas have been handled in each of them.

## Supplementary Material

fwy024_Supplementary_Data

## Footnotes

1 My understanding here is shaped by STS-influenced approaches to the making of legitimacy in scientific practice, for example M Callon, P Lascoumes and Y Barthe, *Acting in an Uncertain World: An Essay on Technical Democracy* (Massachusetts Institute of Technology 2001) or S Jasanoff, *The Fifth Branch: Science Advisers as Policy-Makers* (Harvard University Press 1998).

2 A Wahlberg, ‘A Quackery with a Difference-New Medical Pluralism and the Problem of “dangerous Practitioners” in the United Kingdom’ (2007) 65 Social Science and Medicine 2307–316*.*

3 M Ramsey, ‘Alternative Medicine in Modern France’ (1999) 43 Medical History 286–322, at 320.

4 See for example: J McHale, ‘Legal Frameworks, Professional Regulation and CAM Practice in England and Wales: Is CAM “the special one”?’ in N Gale and J McHale (eds), *Routledge Handbook of Complementary and Alternative Medicine: Perspectives from social science and law* (Routledge 2015).

5 For discussion of the history and contexts of such terminologies, see Ramsey (n 3).

6 Wellcome grant 200380/Z/15/Z.

7 SA Cole and A Bertenthal, ‘Science, Technology, Society, and Law’ (2017) 13 Annual Review of Law and Social Science 351–71; E Cloatre and M Pickersgill (eds), *Knowledge, Technology and Law* (2014).

8 N De Genova, ‘Spectacles of Migrant “Illegality”: The Scene of Exclusion, the Obscene of Inclusion’ (2013) 36 Ethnic and Racial Studies 1180–98; D Cooper, ‘Institutional Illegality and Disobedience: Local Government Narratives’ (1996) 16 Oxford Journal of Legal Studies 255–74; E Cloatre and M Enright, ‘On the Perimeter of the Lawful: Enduring Illegality in the Irish Family Planning Movement’ (2017) 44(4) Journal of Law and Society 471–500.

9 For example: Wahlberg (n 2); McHale (n 4); N Gale, ‘The Sociology of Traditional, Complementary and Alternative Medicine: Traditional, Complementary and Alternative Medicine’ (2014) 8 Sociology Compass 805–22.

10 f (n 2).

11 P Cohen and I Rossi, ‘Le pluralisme thérapeutique en mouvement: Introduction du numéro thématique «Anthropologie des soins non-conventionnels du cancer’ (2011) 2 Anthropologie et Santé (online).

12 P Elzière, ‘Des médecines dites naturelles’ (1986) 4 Sciences sociales et santé 39–74.

13 A Grisoni, ‘De la naturopathie rurale à la santé naturelle: distanciation et assimilation autour de la notion d’espace’ (2012) 8 Nouvelles perspectives en sciences sociales 237; A Grisoni, ‘Sous les pavés, la terre: culte du bien-être et nouveaux métiers: la naturopathie en transformation à la conquête du marché. Doctorat de sociologie, École des hautes études en sciences sociales (EHESS) (2012); A Marcellini and others, ‘Itinéraires thérapeutiques dans la société contemporaine. Le recours aux thérapies alternatives: une éducation à un «autre corps»?’ (2000) 5 *Corps et culture* 1–15.

14 Marcellini and others, ibid; R Gentis, *Leçons du corps* (Paris, Flammarion 1980); Van der Gest and Reynolds Whyte (2003).

15 Eg See the approach taken in 1986 by the Ministry of Social Affairs and Solidarity’s Groupe de Réflections sur les ‘Médecines différentes’. For UK conversations on these issues see for example the House of Lords Science and Technology Select Committee Report (2000) Sixth Report: Complementary and Alternative Medicine, HL: 123; Complementary and Natural Healthcare Council (2013) The First Five Years, London: The Complementary and Natural Healthcare Council.

16 P Pinell, ‘Modern Medicine and the Civilising Process’ (1996) 18 Sociology of Health and Illness 1–16.

17 J Léonard, ‘La médicalisation de l’Etat: l’exemple des premières décennies de la IIIe République’ (1979) 86 Annales de Bretagne et des pays de l’Ouest 313–20.

18 ‘Commission d’enquête sur l’influence des mouvements à caractère sectaire dans le domaine de la santé’. I return below to the very particular entry point of this Commission’s work: that of the link between ‘cultish deviance’ and healthcare.

19 Sénat (2013) *Rapport Fait au Nom de la Commission d’Enquête sur l’Influence des Mouvements à Charactère Sectaire dans le Domaine de la Santé, Tome 2: Procès Verbaux des Auditions’,* Session Ordinaire 2012-2013 N.480: *‘L’autorité du soignant va de pair avec sa légitimité. A partir du XVIIe siècle, sous l’influence des Lumières, le paradigme scientifique est venu fonder la médecine moderne. Peu à peu, cette dimension scientifique a arraché la médecine à la sphère de l’intuition, de la magie, des recettes domestiques et des croyances religieuses, dans lesquelles la maladie pouvait incarner tantôt l’œuvre du démon, tantôt la souffrance rédemptrice. Ne vous trompez pas : les méthodes thérapeutiques illusoires que vous voyez aujourd’hui à l’œuvre sous des aspects folkloriques, orientalistes ou autres nous ramènent à cet ancien paradigme !’*, p 70.

20 art L L4161-1 du Code de la Santé Publique. For comments see for example: Ramsey (n 3); B Lavaud-Legendre, ‘Charlatanisme et droit pénal’ (2008) Les Tribunes de la santé 67–75.

21 art L 4161-5 du code de la santé publique.

22 For examples of relevant case-law, and the broad definition of the offence being applied: Cour de cassation, Chambre criminelle, 28 juin 2016, 15-83.587; Cour de cassation, Chambre criminelle, 9 mars 2010, 09-81.778; Cour de cassation, Chambre civile 1, 16 octobre 2008, 07-17.789; Cour de Cassation, Chambre criminelle, du 21 septembre 2004, 04-80.526; Cour de Cassation, Chambre criminelle, du 2 juin 2004, 03-87.815.

23 Cambrella, *Legal Status and Regulation of CAM in Europe, Part 1: CAM Regulations in the European Countries* (2012) <https://phaidra.univie.ac.at/view/o:291583> accessed 10 May 2018.

24 For a historical review of the pharmaceutical monopoly, see: MD Campion, ‘Les résonances actuelles de la loi de Germinal. Monopole pharmaceutique et exercice illégal de la pharmacie’ (2003) 91 Revue d’histoire de la pharmacie 395–406.

25 art L. 4211-1 Code de Santé Publique.

26 Décret 2008-841.

27 Cour de cassation, criminelle, Chambre criminelle, 20 septembre 2011, 10-83.649; Cour de cassation, criminelle, Chambre criminelle, 22 février 2011, 10-81.359. See also Bureau, 2015.

28 ‘*La Miviludes observe et analyse le phénomène sectaire, coordonne l’action préventive et répressive des pouvoirs publics à l’encontre des dérives sectaires, et informe le public sur les risques et les dangers auxquels il est exposé*.’ Miviludes website, homepage: <http://www.derives-sectes.gouv.fr> accessed 10 May 2018.

29 The spiritual or religious origins of some specific practices, such as naturopathie, have made them particular points of attention of the Miviludes and associated policies—see for example: Grisoni, ‘De la naturopathie rurale à la santé naturelle’ (n 13).

30 JP Chantin, ‘Les sectes en France. Marges et dissidences’ (2000) 66 Vingtième Siècle. Revue d’histoire 67–78; F Champion and M Cohen, ‘Les sociologues et le problème des dites sectes / Sociologists and the “So-called” Sect Issue’ (1996) 96 Archives des sciences sociales des religions 5–15.

31 Miviludes (n 31) Santé et Dérives Sectaires, Paris: Miviludes <http://www.derives-sectes.gouv.fr/missions/actualites/guide-santé-et-dérives-sectaires> accessed 10 May 2018.

32 Loi n.2002-303 du 4 Mars 2002 relative aux droits des malades et à la qualité du système de santé.

33 M Saks, ‘Power and Professionalization in CAM: A Sociological Approach’ in J McHale and N Gale (eds), *Routledge Handbook of Complementary and Alternative Medicines: Perspectives from Social Science and Law* (Routledge 2015) 30.

34 For examples of life-trajectories leading doctors to retrain in acupuncture, see: F Parent, ‘Seuls les médecins se piquent d’acupuncture?’ (2015) N 25 Terrains & travaux 21–38. On homeopathy, see for example Lazarus 2007.

35 O Faure, ‘Une histoire de l’homéopathie’ (1990) 27 Vingtième Siècle, Revue d’histoire 116–17.

36 Ramsey (n 3).

37 See for example J-Y Fagon and C Viens-Bitker, ‘Médecines Complémentaires à l’Assistance Publique-Hôpitaux de Paris : Rapport du Comité ‘d’Orientation’ (2012) <http://cme.aphp.fr/sites/default/files/CMEDoc/cme-10-07-2012_medecines_complementaires.pdf> accessed 23 March 2017.

38 Tribune, 18th March 2018, « L’appel de 124 Professionnels de la Santé cone les “Médecines Alternatives” » <http://www.lefigaro.fr/vox/societe/2018/03/18/31003-20180318ARTFIG00183-l-appel-de-124-professionnels-de-la-sante-contre-les-medecines-alternatives.php> accessed 10 May 2018. The Conseil National de l’Ordre des Médecins responsed on the 22 March : <https://www.conseil-national.medecin.fr/node/2689> accessed 10 May 2018.

39 On issues of epistemological difference, see for example: V Adams, ‘Randomized Controlled Crime: Postcolonial Sciences in Alternative Medicine Research’ (2002) 32 Social Studies of Science 659–90.

40 CR Janes, ‘The Health Transition, Global Modernity and the Crisis of Traditional Medicine: The Tibetan Case’ (1999) 48 Social Science & Medicine 1803–20.

41 Early tensions around the role of doctors in the use of non-biomedical techniques can similarly be found in research on the early days of acupuncture in France (see for example L Candelise, ‘Construction, acculturation etdiffusion de l’« acupuncture traditionaliste française » au XXe siècle’ (2008) 16 Documents Pour l’Histoire des Techniques (online)).

42 Borrowing here from legal consciousness vocabulary and its emphasis on the multiple expressions of law in everyday experiences (see for eg S Silbey and P Ewick *The Common Place of Law* (University of Chicago Press 1998)).

43 Ramsey (n 3).

44 Marcellini and others (n 13).

45 Some professional organisations are critical of such approach, and see it as making them less credible as health professionals, in a country where doctors are not allowed to advertise their own services.

46 Sénat (n 19) 175–85.

47 Example: Cour de cassation, Chambre criminelle, 16 décembre 2014, 14-80.088; Cour de cassation, Chambre criminelle, 9 février 2010, 09-80.681.

48 E Cloatre and M Enright, ‘On the Perimeter of the Lawful: Enduring Illegality and the Irish Family Planning Movement 1972-1985’ (2017) 44(4) Journal of Law and Society 471–500.

49 AC Hoyez, ‘«L’ayurveda, c’est pour les Français». Interroger recours aux soins, systèmes de santé et expérience migratoire’ (2012) 28 Revue européenne des migrations internationales 149–70.

50 Parent (n 34).

51 ibid.

52 Grisoni (n 13).

53 Silbey and Ewick (n 42).

54 A Lazarus and G Delahaye, ‘Médecines complémentaires et alternatives: une concurrence à l’assaut de la médecine de preuves?’ (2007) 15 Les Tribunes de la santé 79–94.

55 Ramsey (n 3).

56 Wahlberg (n 2).

57 For example, an important administrative step for schools and training centres is to see their curriculum recognised by the Répertoire National des Certifications Professionnelles (RNCP). This certification, obtained through a system put in place by the *Ministère du Travail* (ie Ministry of Works and Pensions), bears no relation to the Ministry of Health, or indeed to a recognition of the value of a therapy. Often, however, RNCP recognition is seen and/or presented by healing associations as being significant in relation to the recognition of ‘professions’. The precise role of particular procedures in building legitimacy becomes unclear, as their technicalities are erased behind a broader reference to ‘state recognition’ processes.

58 For example I Robard, *Médecines non-conventionnelles et droit* (Litec 2002).

59 Elzière (n 12).

60 Centre d’Analyse Strategique, (2012) ‘Quelle réponse des pouvoirs publics à l’engouement pour les médecines non-conventionnelles?’ Note d’Analyse 290 <http://archives.strategie.gouv.fr/cas/system/files/2012-10-02-_medecinesnonconvetionnelles-na290_0.pdf Verifier autres initiatives> accessed 10 May 2018; ibid.

61 Sénat (n 19) 206-216.

62
*Les XVIIIe et XIXe siècles avaient vu le passage de « l’Hôtel-Dieu » à « l’Hôpital public ». L’héritage des Lumières a permis l’essor de la médecine scientifique occidentale. Le XXIe siècle risque de générer le glissement de « l’Hôpital public » à « l’autel des gourous ». Les Lumières seraient alors éteintes par l’obscurantisme sectaire que les méthodes thérapeutiques illusoires contribuent à diffuser auprès d’un public avide et de plus en plus influençable !*” Sénat (n 19) 75.

63 ‘*Le relativisme rend donc les superstitions aussi valides que les sciences. La science va même être reléguée au rang de mythe et les critères de rationalité seront désormais présentés comme des contingences d’une culture relative. On tombera alors bien bas !*’ Sénat (n 19) 67.

64 ‘*Depuis le début du dernier tiers du XX^e^ siècle et l’expérience des totalitarismes, on assiste à une remise en cause de la légitimité du fondement scientifique comme si devait être oubliée l’exigence de l’« essai thérapeutique » reposant sur l’emploi du placebo et l’étude dite « prospective, randomisée et en double aveugle ». Les postulats vont ici remplacer les preuves.’* Sénat (n 19) 70.

65 Grisoni (n 13).

66 Parent (n 34).

67 Cohen and Rossi, Le pluralisme thérapeutique en mouvement (n 11).

68 A Guilloux, ‘Évolution de la “tradition” dans l'évolution de la médecine chinoise en France (1860-1980)’ (2011) 5 Revue d'Anthropologie des Connaissances, Société d'Anthropologie des Connaissances 13-40; Grisoni (n. 13)

