# Supplementary material for: Regulating Alternative Healing in France, And the Problem of ‘Non-Medicine’
Source: Med Law Rev. 2018 Jun 27;27(2):189–214. doi: 10.1093/medlaw/fwy024 (PMC6181209; doi:10.1093/medlaw/fwy024)
Supplement: fwy024_Supplementary_Data [file fwy024_supplementary_data.docx]

RÉGLEMENTER LES THÉRAPIES ALTERNATIVES EN France, ET LE PROBLÈME DE LA ‘NON-MÉDECINE’

EMILIE CLOATRE^[[1]](#footnote-1)^

*DISCLAIMER : This translation has not been verified and should not be relied upon – it is provided for reference purposes only. While the Author attests that the translation was undertaken by a professional human translator, the Publishers and Editors have not checked this translation and accept no liability for completeness or accuracy of this translation or the use of this translation for whatever purpose. This translation may be incomplete and inaccurate in whole or in part. If you need to rely upon a translation of this article, a professional human translator should be engaged to supply an accurate translation of the original English. When referencing articles from this journal, please always refer to the original English version, rather than a translated equivalent.*

*NB : Ce texte est une traduction d’un article original publié en Anglais ; elle n’a pas été vérifiée et doit être utilisée uniquement à titre indicatif. Bien que l’auteur atteste que la traduction ait été effectuée par un traducteur professionnel, les Éditeurs n’ont pas vérifié cette traduction, et n’acceptent aucune responsabilité pour les erreurs potentielles de traduction, ni pour l’usage de cette traduction par d’autres. Cette traduction pourrait être incomplète ou erronée, dans son ensemble ou en partie. Si vous souhaitez utiliser une traduction de cette article, nous vous invitons à obtenir une translation certifiée par un professionnel. Toute citation ou utilisation de cet article (ou de tout autre article publié dans cette revue) devra se référer à sa version originale en Anglais plutôt qu’à sa traduction.*

ABRÉGÉ

Cet article examine les ambiguïtés du système légal qui règlemente les soins alternatifs en France et établit les limites en termes de soins médicaux légitimes. Alors que la loi suggère que la prestation de soins thérapeutiques doit être le monopole de professionnels ayant reçu une formation biomédicale, les thérapeutes alternatifs opèrent largement et ouvertement en France. Ils travaillent, cependant, à la limite de la légalité/l’illégalité, organisant souvent leurs activités, individuellement ou collectivement, de façon à limiter la possibilité d’une intervention de l’État. Cette situation crée une certaine précarité, à la fois pour les thérapeutes et, de façon cruciale, pour les patients. Des efforts visant à changer le système sont déployés, mais alors que les guérisseurs s’organisent de plus en plus afin d’obtenir une reconnaissance de l’État, les soins alternatifs occupent un espace politique mal défini: ils ne constituent pas pleinement une question sociale ou politique pour l’État et occupent une position liminaire entre médecine et spiritualité qui bouscule les principes républicains de rationalité scientifique et de laïcité. Ce document examine certaines des tensions qui se trouvent à la jonction entre le droit, la science et la médecine. Il s’interroge sur les raisons pour lesquelles les tensions persistent autour de questions réglementaires cruciales et suggère que, pour aller de l’avant, la science ne soit pas considérée comme le seul élément mediateur servant à définir les limites réglementaires en termes de soins légitimes et illégitimes.

Mots-clés: MAC, thérapies alternatives en France, Droit, science et médecine.

1. INTRODUCTION

Cet article questionne le rôle du droit dans l’élaboration des limites entre les pratiques sanitaires légitimes et illégitimes, ainsi qu’entre le thérapeutique et le non-thérapeutique. Il utilise le cas des thérapies complémentaires et alternatives en France dont la position ambivalente pose la question de ce qui arrive lorsque les patients ont recours à des pratiques de soins qui ne sont pas basées sur des paradigmes médicaux et sont, par conséquent, considérées comme illégitimes par l’État. Il en résulte, en France, au moins deux conséquences notables. La première concerne la zone d’ombre de légalité/illégalité dans laquelle ces pratiques se situent ; elles sont officiellement légales lorsqu’elles sont exercées par des professionnels ayant reçu une formation biomédicale, et co-existent dans les autres cas, de façon plus ou moins précaire, avec le système de santé officiel et réglementé. La seconde consiste à occuper un espace politique mal défini : lorsque les pratiques sont en marge de la médecine, elles cessent également de constituer une question politique pour la plupart des institutions de santé publique. De ce fait, les questions d’ambivalence juridique et de précarité ne peuvent pas être abordées de manière efficace. Pendant ce temps, la légitimité se bâtit et se négocie en marge (et en dépit) des lois, et indépendamment de toute intervention Étatique. Tout au long du présent document, le terme légitimité est défini comme un « processus en cours » et le résultat d’un travail commun entre les régimes légaux et scientifiques. De fait, l’une des particularités du système français réside dans le fait que des tensions persistent entre des conceptions divergentes quant au rôle de la science dans la définition des limites en matière de soins légitimes, et des frontières entre soins légaux et illégaux.^[[2]](#footnote-2)^ L’État français continue, du moins dans ses organisations officielles principales, de fortement s’appuyer sur des paradigmes scientifiques pour établir ces frontières, et résiste à toute mesure qui viserait à s’écarter de cette logique. Dans le même temps, des pratiques quotidiennes remettent en cause ce modèle et appellent à une nouvelle forme de reconnaissance par la loi de nouvelles épistémologies des soins. Les patients, en France comme ailleurs, ont adopté les méthodes de soins alternatifs avec enthousiasme. Toutefois, l’État français progresse plus lentement que d’autres pour organiser le paysage changeant que représente le domaine des soins et pour gérer la fin de ‘l’âge d’or de la biomédecine’.^[[3]](#footnote-3)^Il y a près de 20 ans, Ramsey décrivait la situation comme suit: “la France s’accroche à ce que beaucoup considèrent comme son héritage napoléonique tout en adoptant avec enthousiasme le pluralisme médical”^[[4]](#footnote-4)^. Ceci apporte des indications utiles sur deux questions relatives au droit médical et, de façon plus large, à la recherche juridique: premièrement, comment des pratiques sanitaires opèrent-elles à la limite de la légalite/l’illégalite; deuxièmement, comment les principes de rationalité légaux et scientifiques coopèrent-ils pour définir les limites des soins médicaux. Ces questions sont importantes, à la fois, parce qu’il est aujourd’hui urgent d’aborder le problème des tensions réglementaires qui existent dans le domaine des thérapies alternatives, et parce que bon nombre d’histoires relatent la nature changeante des limites de la loi et de la légitimité dans les pratiques quotidienne de la santé.

L’article examine ces questions en s’intéressant à la façon dont est réglementé le système de santé non biomédical en France, et comment ce dernier fonctionne dans la pratique. Dans le contexte britannique, que les lecteurs connaissent peut-être davantage, ces pratiques sont classées sous l’appellation de Médecines Alternatives et Complémentaires (MAC).^[[5]](#footnote-5)^ En France, une telle appellation n’est pas reconnue,^[[6]](#footnote-6)^ et la terminologie est une question sensible, révélatrice de tensions plus larges concernant la définition de la notion de médecine et de ses limites: l’État a explicitement rejeté le terme “complémentaire” (puisque selon lui la biomédecine ne requiert pas de complément), le terme “alternatif” (une alternative au bien-fondé scientifique étant jugée dangereuse pour le système de santé) de même que le terme “médecines”, la médecine étant synonyme, selon lui, de biomédecine (en effet, on parle souvent, en français, de “médecine” là où le terme de choix en anglais est “biomédecine”). Ces thérapies (certains documents officiels précisant que l’utilisation même du mot “thérapie” leur confère une valeur thérapeutique, ce qui va à l’encontre du discours dominant de l’État) n’ayant pas d’identité médicale, sont difficiles à définir, et par conséquent, à contester. En France, Les MAC sont officiellement appellées “pratiques non-conventionnelles à visée thérapeutique”. Dans le présent article, j’utilise des appellations plus communes en langue anglaise, telles que “thérapies alternatives” ou “systèmes de soins non-biomédicaux”.

Même si cet article focalise sur des pratiques qui sont, de fait, définies par le droit et la Science comme étant non-médicales, il a pour but de contribuer à la recherche en droit médical. En effet, au centre de cet article se trouve la question des frontières du « médical » dans le domaine de la santé, le rôle du droit dans la création de ces frontières, et les implications pour la réglementation des systèmes de soins qui sont en marge de la biomédecine. Outre l’importance conceptuelle et politique, ceci peut également permettre une réflexion sur le droit médical en tant que discipline, et ses limites. Le droit médical ayant fait bon nombre d’efforts pour prendre en compte de nouveaux développements scientifiques et avancées médicales, il semble pertinent d’interroger également le fonctionnement des systèmes juridiques en s’éloignant précisément du biomédical et de sa vision téléologique du système de santé moderne. Ceci permet d’envisager la question des tensions épistémologiques dans la législation qui régit la médecine, et en détermine les frontières.

Après un court résumé des méthodes de recherche, l’article commence par examiner les dilemmes sociaux, politiques et réglementaires soulevés par la question des soins non-biomédicaux en France. Il oppose ces questions au cadre juridique actuellement en place en France, puis compare ce cadre aux pratiques quotidiennes, en mettant l’accent sur les écarts importants qui existent entre ce que la loi prévoit et les pratiques quotidiennes, et en questionnant le sens de cette ambivalence. Enfin, cet article se penche sur la question des tensions qui semblent persister autour d’enjeux réglementaires cruciaux, et suggère que, pour progresser, la science ne soit pas le seul arbitre à décider des limites réglementaires en termes de soins légitimes et illégitimes. Dans un domaine qui est sujet à controverse et à discorde en France, il est important de préciser d’emblée, que l’article ne cherche pas à formuler des affirmations normatives quant au rôle que les pratiques de soin non-biomédicales devraient jouer dans les prestations de santé: au contraire, il propose essentiellement une description critique d’un terrain polémique durable, dont l’ambivalence juridique est devenue une caractéristique dominante et problématique. L’article présente ensuite quelques suggestions sur la manière dont les stratégies réglementaires pourraient être repensées de façon à mieux integrer de telles réflexions critiques, et à redonner à la question son caractère socio-scientifique et socio-juridique grâce à une approche multi-disciplinaire.

II. MÉTHODES

La recherche présentée dans ce document est issue d’un projet plus large qui étudie la relation entre le droit et les médecines traditionnelles et alternatives, en Europe et en Afrique.^[[7]](#footnote-7)^ L’article repose sur les conclusions initiales de l’une des études de cas du projet, la France, provenant d’analyses documentaires; d’une analyse des documents légaux et politiques disponibles sur ce sujet, y compris ceux provenant des autorités publiques et des principales associations thérapeutiques; de la jurisprudence; des débats parlementaires; ainsi que d’entretiens préliminaires avec 15 participants représentant les autorités publiques et les associations thérapeutiques (ces derniers étant tous également des praticiens en activité). L’approbation éthique a été octroyée par le Comité d’Éthique de l’Université du Kent le 1er Mars 2017. Les entretiens semi-directifs utilisés se sont principalement centrés sur l’appréhension des questions juridiques dans le contexte de la pratique quotidienne des participants. Une première liste d’institutions clés, impliquées dans la réglementation de ce secteur d’activités, représentant les professions thérapeutiques, ou participant aux débats spécifiques sur le sujet, fut etablie avant que les participants ne soient contactés par email. Comme ce domaine est extrêmement sensible en France (notamment à cause de la situation juridique décrite ci-dessous), j’ai fait le choix de ne pas enregistrer les entretiens initiaux (à deux exceptions près où les participants étaient particulièrement ouverts) mais plutôt de privilégier la prise de note détaillée. Ceci m’a également permis d’utiliser ces entretiens pilotes pour mieux comprendre les sensibilités et les enjeux liés a ce secteur. En termes de méthodologie, les aspects de la recherche présentés s’appuient sur, et ont pour but de contribuer à, deux champs essentiels de recherche socio-juridique: d’une part, les travaux qui ont porté sur les relations entre le droit et les connaissances scientifiques;^[[8]](#footnote-8)^ d’autre part, les études liées aux pratiques quotidiennes illégales.^[[9]](#footnote-9)^ Ces domaines permettent d’appréhender des questions telles que la la constitution des limites en matière de soins médicaux légitimes, et le rôle des procéssus juridiques et scientifiques dans cette constitution.

1. LES THÉRAPIES NON-BIOMÉDICALES EN FRANCE ET LEUR CONTEXTE SOCIAL

Ces dernières années, les thérapies alternatives ont fait l’objet d’un intérêt accru de la part des chercheurs en droit et en sciences sociales. ^[[10]](#footnote-10)^ Ceci est dû notamment à leur popularité persistante partout dans le monde, une majorité de patients ayant recours à des pratiques autres que celles de la biomédecine pour traiter leurs maux, mais également en raison des problèmes socio-réglementaires qu’elles soulèvent.

En effet, des tensions extrêmement complexes existent entre, d’une part, les préoccupations en matière de sécurité et, d’autre part, la pression en faveur du respect de la liberté de choix des patients. La décision des patients de se tourner vers un traitement alternatif est souvent motivée par des traditions culturelles, des expériences personnelles et une méfiance politique lorsque la biomédecine est vue comme une forme d’entreprise, rendant les décisions dans ce secteur très sensibles. Les débats dans ce domaine sont toujours animés par des tensions sur le rôle de la science dans les traitements médicaux: les défenseurs du savoir scientifique, qui considèrent ce dernier comme moyen principal ou exclusif pour définir les limites du système de santé, s’opposent régulièrement à ceux qui considèrent que le savoir scientifique ne doit pas être le seul à décider ce qui constitue une thérapie légitime. Même si les discussions dans ce domaine sont loin d’être terminées au Royaume-Uni, certaines de ces questions ont été abordées par ce que Ayo Wahlberg appelle la “différenciation par l’intérieur”, un tri au sein des professions MAC, soit grâce à une auto-régulation, soitau moyen de réglementations statutaires entre les pratiques et les praticiens jugées légitimes ou non.^[[11]](#footnote-11)^ Comme il le remarque, cette approche n’est pas sans poser problème, et les tensions continuent de survenir autour de telles légitimités (par exemple, au cours de débats récents concernant la mise à disposition de traitements homéopathiques par le NHS – service de santé national britannique).

La France, quant à elle, offre un contraste intéressant à cette stratégie, et la possibilité d’examiner de plus près, à la fois la question de l’illégalité aux frontières du système de santé, et du rôle de la science dans la co-constitution de la légitimité en droit et en médecine. En France, comme dans d’autres pays, les patients ont fréquemment recours à des thérapies non-biomédicales.^[[12]](#footnote-12)^ Les motifs de ces décisions peuvent être multiples et incluent : une frustration face au système biomédical; une démarche consistant à trouver une forme de soins plus individualisée; un désir de retrouver une approche plus « naturelle » pour la santé,^[[13]](#footnote-13)^ ou des formes de connaissance plus traditionnelles; une volonté politique de s’éloigner des grandes compagnies pharmaceutiques et des aspects capitalistes de la biomédecine; la revitalisation de traditions populaires, locales ou importées, que la biomédecine avait rejétées au cours des années.^[[14]](#footnote-14)^ Cette demande a entraîné ce que certains considèrent comme une prolifération de guérisseurs alternatifs – même si, au vu de la longue tradition de médecine populaire qui existe en France, on est en droit de se demander si cette prolifération en est vraiment une, ou s’il s’agit plutôt d’un regain de visibilité d’un système alternatif de santé qui a toujours existé sous une forme ou une autre. Les specialistes en sciences sociales ont, néanmoins, tendance à s’accorder sur le fait qu’il y a bel et bien un intérêt renouvelé en faveur des pratiques alternatives depuis la période de l’après 1968.^[[15]](#footnote-15)^

Le recours accru aux thérapies complémentaires, ou leur regain de visibilité, depuis les années 1970, a provoqué d’intenses discussions et de vives tensions.^[[16]](#footnote-16)^ Au cours des dernières années, ceci s’est materialisé par des moments de désaccords occasionnels entre une approche disciplinaire, partant du sommet pour aller vers la base, visant à éradiquer toute pratique de soin qui n’est pas “scientifiquement prouvée”, et une pression grandissante (venant essentiellement de thérapeutes) en faveur d’un système de soins pluraliste plus ouvert. En France, le contexte dans lequel ces discussions ont lieu est singulier, notamment, en raison du rôle politique prépondérant que jouent traditionnellement les institutions médicales. Selon les historiens, cette position remonte à l’époque de la Révolution et à l’alliance historique des raisonnements étatique et scientifique/médical dans leur désir mutuel de se détacher de l’Église.^[[17]](#footnote-17)^ Une catégorie particulière de “docteurs d’élite” a depuis occupé des postes influents dans les institutions publiques, et a en partie, modelé les discours officiels déterminant ce qui constitue un système de santé recevable et le rôle de la science au sein de ce système.^[[18]](#footnote-18)^ Prenons comme exemple de moments de tensions occasionnelles, pouvant survenir au sujet des thérapies complémentaires en France, une des conversations officielles récentes datant de 2013 et ayant eu lieu devant une Commission spéciale du Sénat.^[[19]](#footnote-19)^ L’importance de cette Commission ne doit certes pas être surévaluée, elle représente cependant un des rares “moments officiels” de dialogue approfondi sur les soins alternatifs, en France ces dernières années, et par conséquent, une opportunité inhabituelle de voir s’ouvrir une discussion formelle. Les transcriptions des longs débats et des entretiens poussés, sur lesquelles cette investigation repose, donnent la mesure des tensions suscitées par ce secteur d’activités dans la politique française. Voici un exemple, illustré par l’une des personnes interrogées:

“L’autorité du guérisseur va de paire avec sa légitimité. Depuis le 17ème siècle, grâce à l’influence des Lumières, le paradigme scientifique a servi de fondements à la médecine moderne. Progressivement, cette dimension scientifique a éloigné la médecine des domaines de l’intuition, de la magie, des remèdes de grands-mères et des croyances religieuses, selon lesquelles la maladie etait par moments l’oeuvre du diable, et à d’autres une souffrance rédemptrice.

Ne vous méprenez pas: ces thérapies illusoires que vous voyez aujourd’hui à l’œuvre sous des aspects folkloriques, orientalistes ou autres, nous ramènent à ce paradigme ancestral!^[[20]](#footnote-20)^

Si le témoignage de ce participant fut bien accueilli par la Commission, il n’en va pas de même pour ceux dont le regard sur les thérapies alternatives est moins critique, notamment ceux qui proposent d’entreprendre d’éventuelles recherches en vue de déterminer si certaines de ces thérapies pourraient contribuer à la prise en charge des patients. Les débats engendrés devant cette Commission offrent d’autres aspects notables. Par exemple, l’importance de la “révolution scientifique” et la nécessité de ne pas en abandonner les enseignements, tels que ceux mentionnés ci-dessus, sont des aspects qui y figurent de façon répétée. Les discussions relatives à la notion de liberté thérapeutique ont également leur place: la liberté thérapeutique est considérée comme un élément important, qui ne peut, toutefois, être exercé que dans le cadre de thérapies reconnues (à savoir, ici, la biomédecine). Enfin, le rejet systématique des médecines non-occidentales nous rappelle pourquoi la question de la rationalité en médecine est également chargée de significations culturelles, y compris (post)coloniales.

1. CADRE JURIDIQUE: DÉFINIR LES LIMITES DU LÉGAL ET DE L’ILLÉGAL DANS LES SECTEURS THÉRAPEUTIQUES

Cette sous-partie se penche sur la question du cadre légal qui régit les thérapies complémentaires en France, et autour duquel des discussions sont actuellement en cours. Elle attire, par ailleurs, l’attention sur trois caractéristiques essentielles: tout d’abord, par principe, seuls les docteurs en médecine ou professions autorisées (tels que les sages-femmes, les dentistes et les infirmiers, et tous dans le respect de limites établies) peuvent pratiquer un acte de soin; deuxièmement, seuls les pharmaciens sont, en principe, aptes à vendre des produits considérés comme “médicinaux”; troisièmement, toutes thérapie alternative peut faire l’objet d’une surveillance de la Miviludes (Mission Interministérielle de Vigilance et de Lutte contre les Dérives Sectaires), une agence responsable du contrôle des sectes et des cultes. Ces trois aspects créent un espace limité pour les thérapies complémentaires qui sont, lorsque la loi le permet, subordonnées à la biomédecine et confinées aux professions médicales.

1. **Exercice illégal de la médecines et les limtes professionnelles du soin**

Depuis 1804, et à travers divers ajustements au cours des années, tout acte visant à “traiter ou diagnostiquer” est illégal s’il n’est pas réalisé par un médecin qualifié.^[[21]](#footnote-21)^ Dans un tel cas, la peine encourue peut aller jusqu’à 2 ans de prison et/ou une amende de 30 000 euros.^[[22]](#footnote-22)^ Les désignations “traiter” et “diagnostiquer” ont été interprétées au sens large. Par exemple, prétendre qu’une intervention spécifique est curative, quelle que soit son efficacité potentielle, peut constituer une pratique illégale de la médecine. Concrètement, cela signifie que la plupart des thérapies complémentaires telles que l’herboristerie populaire, la médecine chinoise traditionnelle, la naturopathie ou l’acupuncture sont officiellement illégales si elles sont pratiquées par des non-médecins.^[[23]](#footnote-23)^ Cette approche de la réglementation par la criminalisation n’est pas propre à la France, certains systèmes comparables du sud de l’Europe utilisant des techniques prohibitives similaires.^[[24]](#footnote-24)^ Cependant, cette position devient de plus en plus inhabituelle dans un contexte réglementaire changeant, et fait l’objet de pressions croissantes pour revoir l’étendue du monopole dont bénéficient les médecins dans la prestation des services de santé. Comme je l’illustre ci-dessous, l’application pratique de la loi n’est pas aussi stricte ou marquée que le texte le laisse entendre – même si cela n’en est pas moins problématique ou inquiétant du point de vue des thérapeutes alternatifs.

**B, L’exercice illégale de la pharmacie et la vente des produits thérapeutiques**

Il existe en parallèle et en complément de ce cadre légal un monopole comparable sur les ventes de produits médicinaux. Deux séries de réglementations sont utiles ici: celles qui concernent les professions, et celles qui concernent les produits. L’exercice illégal de la pharmacie signifie que nul n’a le droit de vendre des médicaments, excepté un pharmacien qualifié, et ce dans l’enceinte d’une pharmacie.^[[25]](#footnote-25)^ La définition de ce qui constitue un médicament est large et la frontière entre ce qui est considéré médicinal ou non médicinal est floue. Tout produit revendiquant un effet sur la santé constitue un médicament. La position liminaire des plantes médicinales et des produits à base de plantes rend leur réglementation en tant que produits particulièrement complexe. Voici, en résumé, les conditions de vente qui s’y rapportent: sur le principe, seuls des pharmaciens qualifiés peuvent, depuis 1941, vendre des plantes médicinales à l’état brut, ou des produits médicinaux à base de plantes.^[[26]](#footnote-26)^ Les herboristes qualifiés constituaient jusqu’alors l’exception, mais le diplôme correspondant à cette formation disparût à la suite d’une loi promulguée en 1941 (par le gouvernement de Vichy). Pour faire exception à cette règle, une liste de plantes pouvant être vendues librement fut créée en 2008, et comprend désormais 148 plantes.^[[27]](#footnote-27)^ Les conditions de vente de ces plantes sont toujours restreintes s’il ne s’agit pas d’un pharmacien qualifié. Dans ce cas, et à quelques exceptions près, elles ne doivent ni être mélangées ni porter de message indiquant quelque propriété médicinale que ce soit. Seuls les pharmaciens peuvent prodiguer des conseils, par exemple, quant à la façon d’administrer une plante et quelles affections cette dernière peut traiter. Quiconque prodiguant un conseil sur ces plantes (y compris les herboristeries ou les magasins vendant des produits de santé) doit être un(e) pharmacien(ne) qualifié(e) sous peine de pratique illégale de la pharmacie. Des restrictions similaires existent pour les produits à base de plantes manufacturés: ‘les compléments alimentaires’ peuvent être vendus par des personnes n’étant pas pharmaciens; en revanche, l’étiquetage ou le conseil prodigué sur ces produits peuvent en faire un médicament et de ce fait, mettre le fournisseur de ce produit dans une situation de pratique illégale de la pharmacie.^[[28]](#footnote-28)^

Le concept d’illégalité présent autour des pratiques, des professions et des produits thérapeutiques, ou autour de ceux qui s’en réclament ou s’y apparentent, est sans aucun doute déterminant pour le développement officiel des thérapies alternatives. La loi n’autorise, du moins sur le papier, que les professionnels et les produits du biomédical à répondre aux besoins sanitaires de la population. Les autres ne sont pas autorisés à intervenir dans ce domaine, quelle lque soit la demande des patients, sous peine d’être poursuivis en justice. Je reviens, ci-dessous, sur les contrastes entre ce système supposément strict, et sa mise en pratique.

1. **Sectes, thérapies alternatives et surveillance étatique**

Une troisième caractéristique du système français, liée autant à l’approche de la religion en France qu’à la question de la médecine, réside dans le fait que la Miviludes surveille certains aspects (et abus) des médecines alternatives. La Miviludes représente une particularité de l’environnement réglementaire français et alimente un débat plus vaste sur la laïcite et la liberté de religion qui dépasse, en partie, le cadre de cet article. Créée au milieu des années 1990 (sous un autre nom et avec une structure légèrement différente), son but est de surveiller et de contrôler ce que l’on appelle les “dérives sectaires”.^[[29]](#footnote-29)^ S’agissant de sa structure, la Miviludes consiste en une mission interministérielle gérée par un ensemble de membres permanents organisés en quatre sous-équipes qui se concentrent respectivement sur la surveillance des sectes dans les domaines de la jeunesse et et de l’éducation; la sécurité; le travail et l’emploi; et la santé (ce dernier groupe étant constitué de seulement deux membres à temps plein). Les groupes sont coordonnés par un président et un secrétaire général, et secondés par des services administratifs. Les actions menées par la Miviludes et leurs directions générales sont également définies par des discussions entre les ministères, au sein d’un comité exécutif comptant des membres de chaque ministère concerné, et d’un conseil d’orientation composé d’acteurs publics nommés par le bureau du premier ministre (y compris des parlementaires, par exemple) et de représentants d’associations-clés, essentiellement liés aux secteurs de la jeunesse et de l’éducation. Bien qu’une étude détaillée de la Miviludes dépasse le cadre de cet article, il convient de souligner, par le biais de cette brève description, qu’elle fonctionne à la fois comme agence de surveillance et comme espace de décisions politiques plus générales dans lequel sont négociées des questions complexes sur la relation entre l’État et les pratiques religieuses.

D’une certaine façon, la raison expliquant l’intervention de la Miviludes dans le domaine des soins alternatifs est simple, même si elle est particulière dans ses effets et sa structure: l’hypothèse sous-jacente consiste à penser que la santé est un point de départ significatif pour les chefs de file de cultes cherchant à recruter de nouveaux adeptes.^[[30]](#footnote-30)^ On invoque comme l’un des facteurs possibles la vulnérabilité des patients qui, une fois approchés, peuvent tomber sous une emprise psychologique excessive. De tout temps, les sectes ont déployé des stratégies thérapeutiques ou pseudo-thérapeutiques ; leurs chefs de file se sont souvent présentés comme ayant également des pouvoirs guérisseurs qui permettraient à leurs adpetes de vivre une vie plus saine et plus riche. Bien évidemment, le contexte spécifique de la laïcité en France joue, par ailleurs, un rôle significatif dans l’émergence d’un certain discours et de pratiques institutionnelles autour de la question des cultes.^[[31]](#footnote-31)^ Même si la Miviludes estime qu’elle n’a pas pour rôle de contrôler l’inorthodoxie thérapeutique, voire même de surveiller les cas de pratiques illégales de la médecine en tant que telles, elle enquête et/ou intervient lorsqu’une situation se présente où l’influence du praticien a un impact sur le jugement du patient et se traduit par une emprise excessive.^[[32]](#footnote-32)^

Il est clair pour les représentants de la Miviludes qu’ils n’ont pas un rôle de santé publique ni de surveillance des praticiens qui n’entrent pas dans la catégorie de cette forme de contrôle mental plus étroite et plus extrême que constituent les dérives sectaires. Cependant, le fait que leur responsabilité officielle inclut la surveillance des praticiens fait d’eux des acteurs relativement centraux dans ce qui semble par ailleurs être un vide politique, comme je le décris ci-dessous. Souvent, lorsqu’au Parlement, des questions sont posées pour assouplir la loi actuelle sur les pratiques médicales, de façon à inclure les thérapies alternatives, ou que l’on s’interroge sur la reconnaissance/réglementation officielle des MACs, les réponses officielles indiquent simplement que l’activité des praticiens alternatifs est réglementée, à la fois, par la loi sur l’exercice illégal de la médecine, et sous la surveillance de la Miviludes. La symbolique de ce discours est représentative, même si, en pratique, les herboristes populaires ou les thérapeutes de médecine chinoise ont peu de contacts avec la Miviludes, qui ne s’intéresse pas fondamentalement à eux, sauf dans les cas de dérives sectaires.

1. **Quelques exceptions limitées, au sein du cadre biomédical**

Avant de s’intéresser à l’ambivalence du système réglementaire en pratique, mentionné ci-dessus, et à quelques-uns de ses effets, il convient d’indiquer, au moyen d’exceptions, les espaces que la loi crée pour les thérapies alternatives. Certes, celles-ci ne changent pas foncièrement les fondements biomédicaux du système, mais elles créent cependant une certaine ambivalence. Arrêtons-nous brièvement sur ces deux séries d’exceptions: premièrement, l’émergence de l’ostéopathie et de la chiropraxie, premières professions alternatives reconnues par l’État français. Deuxièmement, la possibilité donnée aux praticiens ayant une formation biomédicale de pratiquer des thérapies alternatives, ainsi que leur présence dans certains espaces propres à la biomédecine, tels que les pharmacies.

1. *L’émergence de professions : ostéopathie et chiropraxie*

L’ostéopathie et la chiropraxie font, depuis 2002, l’objet d’une réglementation légale et sont reconnues comme professions de santé.^[[33]](#footnote-33)^ Les séances d’ostéopathie ou de chiropraxie sont remboursées par le système de sécurité sociale ou par les mutuelles (à des taux divers selon les régimes de mutuelles et selon les circonstances) dès lors qu’elles sont prescrites par un médecin. Les raisons expliquant l’émergence de ces deux professions en particulier ne sont pas tout à fait claires: il n’y a, en effet, aucune raison évidente, conceptuelle ou tangible, pouvant expliquer le statut de ces deux professions dans un système par ailleurs fermé aux pratiques non-biomédicales. Plusieurs facteurs semblent cependant avoir participé à cette reconnaissance. Premièrement, certains participants à ce projet avancent que l’une des raisons de cette reconnaissance réside dans le fait que la loi sur l’exerice illégal de la médecine, en ce qui concerne l’ostéopathie et la chiropraxie, avait cessé d’être dûment appliquée avant que l’État ne règlemente ces professions de façon officielle. Deuxièmement, ces pratiques sont vraisemblablement moins en conflit direct avec le monopole des médecins, et empiètent plus clairement sur le terrain des kinésithérapeutes (qui ont traditionnellement moins d’influence politique que les médecins). Troisièmement, les ostéopathes et les chiropracteurs constituent, depuis longtemps, une profession solidement organisée, offrant au législateur l’assurance du respect des normes professionnelles. En effet, ils furent également l’une des premières professions MAC à être réglementées, sur le plan légal, au Royaume-Uni.^[[34]](#footnote-34)^ Enfin, il convient de noter que, si le modèle fourni par les ostéopathes et les chiropracteurs est souvent considéré comme un modèle à suivre par d’autres professions cherchant à être reconnues – aspect sur lequel je reviens ci-dessous – il n’échappe pas à la controverse. Pour certains, la reconnaissance professionnelle est arrivée trop tôt, avant que des structures solides ne soient en place pour pouvoir vérifier et contrôler les écoles et les diplômes. Ainsi, jusqu’à récemment, l’augmentation rapide du nombre d’ostéopathes aurait résulté en des normes hétérogènes au sein de la profession.

1. *Alternatives « à l’intérieur »*

Deuxièmement, les acteurs et les institutions reconnus pas la biomédecine peuvent, jusqu’à un certain point, adopter des épistémologies alternatives même si la loi fait en sorte de soigneusement écarter du système de santé ceux qui n’ont pas de formation biomédicale. Ceci s’applique aux médecins, aux pharmaciens et, d’une certaine manière, à l’industrie pharmaceutique. De fait, la réglementation actuelle des traitements et des diagnostics est basée sur la limite entre ceux qui sont autorisés (les professionnels du biomédical ayant des qualifications) et ceux qui ne le sont pas, plutôt que sur une interdiction de pratiques spécifiques. Les docteurs (et, dans une certaine mesure, d’autres professions de santé comme les dentistes, les infirmiers ou les sages-femmes) sont ainsi en mesure de proposer également une forme de thérapies alternatives. Il est relativement commun, par exemple, que les médecins proposent l’acupuncture ou l’homéopathie à leurs patients,^[[35]](#footnote-35)^ ces deux pratiques ayant, en effet, une longue histoire dans les cercles médicaux.^[[36]](#footnote-36)^ Lorsqu’un traitement est pratiqué ou prescrit par un médecin, il est partiellement remboursé par le système de sécurité sociale (et souvent complété par les mutuelles), même si des questions concernant le taux de remboursement de certains actes subsistent et les controverses liées à une telle couverture sociale apparaissent régulièrement. De nombreuses universités proposent des diplômes de développement professionnel pour les médecins souhaitant suivre une nouvelle formation, y compris dans le domaine des thérapies complémentaires.^[[37]](#footnote-37)^ Lorsque les médecins pratiquent ces thérapies alternatives, ils restent sous la surveillance de l’Ordre des Médecins, et doivent s’assurer qu’ils continuent de proposer les prestations de soins que leurs homologues considèrent suffisantes et adaptées, mais la pratique de thérapies alternatives en soi est autorisée. Dans le cas de pratiques établies comme l’acupuncture et l’homéopathie, les médecins expérimentés peuvent être extrêmement populaires auprès des patients, et leur pratique extrêmement rentable. En outre, des espaces pour les thérapies complémentaires ont été introduits dans les hôpitaux.^[[38]](#footnote-38)^ Ceci ne veut pas dire que de telles pratiques ne sont pas controversées, même au sein du monde médical. Par exemple, le 18 mars 2018, 124 médecins ont publié une lettre ouverte dans le Figaro, journal (de tendance politique conservatrice) largement diffusé, dans lequel ils exigeaient une approche plus stricte envers leurs homologues pratiquant l’homéopathie et autres “fausses médecines” (comme ils les appellent): des pratiques qui ne sont “ni scientifiques ni éthiques mais irrationnelles et dangereuses".^[[39]](#footnote-39)^ La lettre réclame une série de changements et exige que l’Ordre des Médecins prenne des sanctions contre les médecins qui pratiquent de telles thérapies, ainsi que la fin de la couverture sociale pour de telles pratiques et l’arrêt des formations pour de telles techniques dans les facultés de médecine. Il reste à voir si une telle lettre entraînera des changements et des discussions autres que celles générées sur les réseaux sociaux. Dans l’ensemble, malgré quelques périodes de controverse, la situation en France reste, pour le moment, ambivalente et ironique, dans le sens où seuls les médecins peuvent pratiquer de façon légale autre chose que la médecine.

De façon similaire, les pharmaciens restent en mesure de vendre des remèdes alternatifs, médicinaux ou à base de plantes. La plupart des pharmacies en France proposent également à la vente une gamme étendue de produits homéopathiques, phytothérapiques ou, plus rarement, de plantes médicinales. Ces produits sont extrêmement populaires et permettent à une large industrie de produits homéopathiques et médicinaux à base de plantes de prospérer – tant que leur approvisionnement est contrôlé par des pharmaciens, et limité à l’espace de vente des pharmacies.

Ici, le système légal autorise une forme singulière de pluralisme “de l’intérieur” propre à créer une certaine ambivalence quant à l’approche de l’État sur les thérapies alternatives. D’un côté, comme le soulignent régulièrement les autorités publiques, des opportunités sont créées pour répondre aux demandes des patients qui recherchent quelque chose de plus ou d’autre que la biomédecine. Mais, en même temps, proposer de telles alternatives au sein de la biomédecine suggère qu’elles ne sont, en fait, pas complétement “alternatives”. De fait, le degré de changement réellement exigé par les patients, lorsqu’ils recherchent des pratiques médicales alternatives, semble être sous-estimé : par exemple, il est probable que les patients qui se tournent vers des praticiens alternatifs le fassent précisément afin d’échapper à l’institution biomédicale. Parallèlement, la logique qui veut que quiconque, pratiquant dans le cadre d’un système alternatif de pensée et de soin, ait également reçu une formation en biomédecine, est tout aussi équivoque: d’une part, l’État a pour but de garantir que de tels systèmes de soin sont offerts par des professionnels opérant dans le respect des normes appropriées, grâce à une formation qui permet de limiter les risques de diagnostics erronés, et qui sont également soumis à une surveillance de la part d’organes de réglementation professionnelle reconnus. De ce point de vue, il est judicieux d’autoriser les médecins à pratiquer d’autres thérapies, comme il l’est d’attendre de ceux qui pratiquent d’autres thérapies d’être aussi formés en biomédecine. D’un autre côté, il ne semble pas si logique d’escompter que quiconque pratiquant selon les préceptes de la médecine chinoise, si fondamentalement différents de ceux de la biomédecine, doive recevoir une formation, en amont, basée sur les principes contrastants voire incompatibles de la biomédecine.^[[40]](#footnote-40)^ Une logique réglementaire est également mise en avant pour justifier le confinement de l’“alternatif” à l’intérieur de l’institution: notamment, les médecins étant déjà supervisés par l’Ordre des Médecins, il existe une forme de contrôle réglementaire de leurs activités, et un moindre risque de pratiques problématiques. Si cet argument est important sur le plan juridique, il n’y a, en principe, aucune raison qu’un tel organisme professionnel ne soit pas développé pour d’autres professions. En effet, c’est la voie qu’on a choisie de suivre au Royaume-Uni pour réguler les professions alternatives “de l’intérieur”. De la même façon, la possibilité d’accéder à des traitements “plus doux” ou “plus naturels” dans les pharmacies répond, dans une certaine mesure, à une demande des patients. Cependant, il s’agit d’une réponse très limitée qui néglige certaines composantes sociales essentielles: premièrement, insistons sur le fait qu’en recherchant des options naturelles, les patients sont probablement en quête d’une alternative à la dimension clinique proposée par le système biomédical – ce qui expliquerait leur intérêt pour des magasins de santé au détriment des pharmacies. Deuxièmement, le fait que, l’opposition politique à la biomédecine qui est en partie responsable du développement des mouvements de thérapies alternatives, constitue également une opposition à sa dimension industrielle. Enfin, d’un point de vue analytique, subordonner les pratiques alternatives à la biomédecine rappelle l’argument classique qui consiste à penser que la biomédecine, en tant qu’institution, est une entreprise colonisatrice – tout comme le droit d’ailleurs.^[[41]](#footnote-41)^ On entend par là que les traditions thérapeutiques anciennes importées (telles que la médecine tibétaine ou chinoise), ou les traditions populaires qui ont historiquement vu le jour parallèlement aux élites biomédicales, sont de plus en plus conditionnées par la biomédecine, et ne peuvent être pratiquées que par ceux dont les connaissances proviennent du biomédical.^[[42]](#footnote-42)^

De manière générale, le système français présente un certain paradoxe qui est totalement intégral à la loi elle-même. Au sein de la sphère biomédicale, les thérapies alternatives peuvent prospérer, et sont à la fois populaires et, souvent, lucratives pour les médecins, les pharmaciens et l’industrie pharmaceutique. Parallèlement, lorsque les thérapies alternatives opèrent en dehors des limites de la biomédecine, exception étant faite des ostéopathes et des chiropracteurs, elles sont considérées juridiquement comme problématique et, de fait, criminelles.

1. NÉGOCIER LA PRATIQUE À LA FRONTIÈRE DE LA LÉGALITE THÉRAPEUTIQUE

Le système juridique qui régit la médecine alternative en France, et le discours plus large des autorités publiques qui le soutient, apparaît ambivalent: d’un côté, une grande méfiance prohibitive à l’égard des thérapeutes alternatifs; de l’autre, l’ouverture d’espaces permettant aux pratiques alternatives de se développer dans le contexte de la biomédecine, sous son contrôle et par le biais de ses agents. Comme c’est souvent le cas, l’expérience vécue est loin de s’aligner sur le contenu juridique du système. Dans la pratique, les thérapies alternatives sont, en France, communément usitées, relativement visibles et très populaires. Rien ne montre que la loi en limite l’usage – même si elle a indéniablement une incidence sur les expériences de chacun: d’après le contenu de la loi, ces thérapies se pratiquent à la limite de la légalité/de l’illégalité. Certaines activités sont totalement illégales, mais sont tolérées, alors que d’autres trouvent des moyens de s’adapter à la loi pour rester formellement légales. Si la loi n’a en rien limité l’offre ou la demande, elle a façonné la manière dont les pratiques alternatives sont formulées et les conditions de leur usage. À travers leurs activités, les praticiens ont développé des techniques normatives différentes de celles décidées par l’État. Ils se sont organisés afin d’éviter des situations juridiques critiques et d’œuvrer vers un changement de positionnement social, à la fois, de leur pratique et, à terme, de la loi. Dans le même temps, ce système de négociation est loin d’être satisfaisant du point de vue des thérapeutes eux-mêmes, des autorités publiques voire des patients, d’autant plus que ces pratiques fonctionnent au sein d’un fossé politique relatif dans lequel les instances et les modes de réflexion stratégiques sont extrêmement limités et essentiellement centrés sur le rôle prédominant que joue la science pour résoudre les polémiques actuelles. Dans cette section, j’explore quelques-unes de ces questions et le sens du « non-médical » en termes juridiques,^[[43]](#footnote-43)^ avant de me pencher sur la place de la science dans ces conversations. Je soutiens l’idée que, contrairement à ce que les pratiques politiques ont laissé entendre jusqu’à présent en France, les questions juridiques complexes qui sont en jeu, ne peuvent être résolues uniquement par le biais de la science. Elles mettent en lumière des tensions socio-culturelles profondes qu’il serait préférable d’aborder grâce aux outils et aux connaissances dont disposent les sciences sociales.

1. **Recours aux thérapies alternatives en France : de restrictions légales à usage généralisé**

Malgré le cadre juridique en place qui suggère que seuls les médecins sont aptes à traiter les patients, on trouve des thérapeutes alternatifs en France tout aussi facilement qu’ailleurs.^[[44]](#footnote-44)^ Par exemple, tout patient, en France, recherchant un naturopathe, un praticien en médecine chinoise traditionnelle ou un magnétiseur, peut les trouver sans problème en ligne, dans les pages jaunes (sous le label générique « autres professions de santé ») ou tout simplement sur des plaques d’immeubles ou sur les boites aux lettres.^[[45]](#footnote-45)^ De nombreux thérapeutes font une promotion proactive de leurs services dans les journaux locaux ou par le biais de dépliants qu’ils laissent dans les lieux publics.^[[46]](#footnote-46)^ La présence de ces praticiens est si manifeste qu’il est courant que les patients n’aient pas conscience de l’illégalité ou quasi-illégalité dans laquelle ces derniers pratiquent.

Certains thérapeutes alternatifs travaillent parfois avec d’autres professionnels de la santé (ayant des qualifications biomédicales). Par exemple, en plus du personnel hospitalier (médecins et infirmiers) qui propose des soins et des techniques alternatifs, certains hôpitaux utilisent les services de thérapeutes externes, qui ne sont pas nécessairement eux-mêmes des professionnels de la santé. Prenons l’exemple des hôpitaux de Paris qui offrent des services de sophrologie, de shiatsu et des éléments de médecine chinoise traditionnelle à leurs patients pour en explorer les possibles bienfaits – cette décision ayant rendu furieuse la commission du Sénat mentionnée ci-dessus.^[[47]](#footnote-47)^ Les herboristes interrogés dans le cadre de ce projet ont déclaré travailler avec des hôpitaux locaux ainsi que des médecins, et offrir occasionnellement une formation aux praticiens de la santé désireux de mieux comprendre les remèdes à base de plantes. Si cette collaboration avec le personnel du domaine biomédical n’est pas exceptionnelle, elle n’est pas non plus caractéristique de toutes les perceptions qu’ont les professions biomédicales des thérapeutes alternatifs : pour certains, les soins alternatifs voire complémentaires sont encore à peine plus qu’une perte de ressources pour des patients trop crédules. Enfin, les thérapeutes alternatifs sont des professionnels à leur compte qui doivent payer des impôts et peuvent bénéficier d’une assurance professionnelle et certaines mutuelles remboursent désormais certaines de leurs consultations. En d’autres termes, malgré la précarité de leur activité, ils ne constituent pas un ensemble d’acteurs discrets, qui pratiqueraient clandestinement, ce qui suggère au moins un degré de tolérance de la part de l’État, et une ambivalence de leur positionnement socio-juridique. Bien évidemment, tout comme ailleurs, l’éventail de thérapies et de thérapeutes auxquels les patients peuvent avoir recours, est extrêmement varié. Les herboristes, naturopathes, guérisseurs chinois, acupuncteurs non-médecins, magnétiseurs et sophrologues cohabitent avec des mouvements plus obscures de thérapies émergeantes. Si certains ne correspondent pas aux descriptions les plus méfiantes données par les responsables publics, l’image du « guérisseur dangereux » qui anime le système juridique n’est pas complètement imaginaire. Outre les pratiques et les professions bien établies qui partagent un système d’éthique et de pratique calqué sur celles des professions de santé en place et qui souhaitent se positionner sur l’échiquier d’un système de santé plus large, des mouvements plus obscurs émergent et affirment de façon plus radicale que tel individu ou telle technique a des pouvoirs miraculeux, poussant les patients à délaisser le système de santé établi.

De même, certains individus s’auto-proclaments guérisseurs sans pour cela présenter la moindre preuve de formation ou d’attachement professionnel ou épistémologique, et constituent un nouvel élément de « charlatanisme » à la fois pour les thérapeutes alternatifs et l’État. Établir une stratégie réglementaire en présence de tels personnages peut s’avérer problématique: cette situation met en évidence la nécessité de définir les frontières de la légalité dans le domaine sanitaire, et de voir les autres praticiens s’engager et participer à l’établissement de ces frontières.

1. **Les limites de l’application de la loi**

De manière générale, la pratique de soins alternatifs en France est à la fois commune et visible, allant bien au-delà des espaces que lui réserve la loi. A première vue, ceci est clairement en opposition avec ce que la loi semble suggérer, à savoir que seuls les professionnels de la santé ayant reçu une formation biomédicale peuvent faire fonctionner le système de santé. Au moins deux paramètres sont ici à prendre en compte. Le premier montre que l’application de la loi dans ce domaine est difficile, comme l’évoquent les acteurs publics ayant pris part à cette recherche. Même si la loi semble catégorique lorsqu’elle affirme que seuls les médecins sont en mesure de traiter ou de diagnostiquer un patient, et même si, comme nous l’avons vu, les tribunaux ont élargi ces modalités de manière à inclure les affirmations concernant la valeur thérapeutique d’un acte donné, il reste très complexe de fournir des preuves. Souvent, cet acte doit être constaté, ce qui est difficile à réaliser lors de consultations privées. De plus, et j’y reviens ci-dessous, les praticiens sont attentifs à la manière dont ils s‘expriment pour ne pas être en opposition à la loi.

En pratique, cela signifie que l’application de la loi a tendance à focaliser sur les pratiques où un tel espace de négociation est plus limité, et où il est plus facile de qualifier de médical la nature de l’acte en soi. L’acupuncture en est un exemple et, de fait, représente l’une des activités pour lesquelles les thérapeutes sans formation médicale sont régulièrement poursuivis : introduire une aiguille dans la peau d’une personne est, en soi, un acte médical/thérapeutique et il est illégal pour toute personne non autorisée (les médecins, les dentistes, et plus récemment les sages-femmes) de recourir à cet acte.^[[48]](#footnote-48)^ Par ailleurs, la jurisprudence suggère que la loi a été appliquée dans des cas de tromperie et d’abus, ayant été bien au-delà des pratiques quotidiennes de la plupart des thérapies alternatives, et s’étant étendu sur de longues périodes de temps. Les participants ont cependant insisté sur le fait que les poursuites sont dans la pratique extrêmement imprévisibles et qu’il y a toujours un degré d’incertitude sur le commencement des poursuites (même lorsque les accusations sont retirées plus tard). Selon eux, les poursuites arrivent souvent par vagues et dépendent de circonstances locales spécifiques souvent liées aux convictions ou aux inquiétudes de procureurs locaux et de représentants locaux de l’Ordre des Médecins, ou relatives aux activités d’une sous-région spécifique. Cette sensation d’imprévisibilité ajoute, dans la pratique, au sentiment de précarité sur lequel je reviens ci-après.

1. **Négotier la tolérance**

Parallèlement à cette difficulté d’appliquer la loi en tant que telle, et comme c’est communément le cas dans des situations d’illégalité persistante,^[[49]](#footnote-49)^ les thérapeutes ont également trouvé des moyens subtils de contourner la loi. Ils ont appris, collectivement et individuellement, grâce à leurs interprétations vernaculaires de la loi et à l’aide d’avocats spécialisés, à mettre en oeuvre des techniques leur permettant de continuer à respecter la loi. A titre d’exemple, ils font souvent très attention au vocabulaire qu’ils utilisent pour décrire leurs actions, pour éviter de se présenter de façon trop « médicale » voire « thérapeutique ». ^[[50]](#footnote-50)^ Les conseils prodigués à leurs patients relèvent plutôt du « conseil de vie » que de « l’ordonnance ». Certains préfèrent ne pas du tout donner de conseil écrit.^[[51]](#footnote-51)^ De plus en plus, les thérapeutes organisent et partagent leurs expériences et se soutiennent pour répondre aux exigences de la loi. Au fil des années, des organisations professionnelles se sont développées offrant des registres professionnels, des conseils d’ordre juridique et des codes de pratique pour certains thérapeutes.^[[52]](#footnote-52)^ Les naturopathes, les herboristes, les praticiens de médecine chinoise traditionnelle, se sont organisés en fédérations, syndicats ou associations qui défendent leurs intérêts professionnels, et s’efforcent de développer un système de réglementation parallèle qui, bien que non reconnu par l’État, offre un cadre réglementaire fantôme au sein duquel ces professions peuvent fonctionner. En outre, les programmes de formation incluent également un élément « légal ». Individuellement et collectivement, ces professions émergentes donc ont appris à faire en sorte que leur pratique puisse coexister avec la loi.^[[53]](#footnote-53)^ La négociation de la légalite/de l’illégalite est une entreprise professionnalisée et collective.

Les techniques de négociation relatives au droit et le contournement de ce dernier sont permis et maintenus grâce à des facteurs externes qui expliqueraient, selon les participants, pourquoi il existe, dans la pratique, un degré apparent de tolérance de la part de l’État. L‘une des hypothèses communément avancée par les participants est qu’un éventail d’intérêts, au-delà de ceux des thérapeutes, profitent des pratiques de soins alternatifs, ce qui ajoute à la nécessité de renforcer la loi, particulièrement au niveau local. Il est, par exemple, fréquemment affirmé que, les autorités locales bénéficient financièrement de ces activités : même s’il est difficile de définir avec certitude le rôle que cet aspect joue dans l’application de la loi et/ou la tolérance (en particulier si l’on a à l’esprit la nature complexe et à plusieurs niveaux de ‘l’État ‘), une économie substantielle s’est développée grâce aux traitements alternatifs, entraînant des ramifications locales. À titre d’exemple, les salons et les conférences sur les thérapies alternatives sont des entreprises extrêmement rentables qui, inévitablement, regroupent tout un ensemble d’intérêts financiers. Au-delà de tels enjeux économiques, des participants soutiennent que les autorités locales profitent des thérapeutes alternatifs pour assurer certaines prestations de santé : le manque de médecins est un problème récurrent en France ces dernières années, particulièrement dans les zones rurales où les communautés peuvent se retrouver sans aucun soutien médical ou aucune prestation de soins (les ‘déserts médicaux’). Par conséquent, on considère que les praticiens alternatifs peuvent « combler le vide » en proposant un service de santé local, ce que les autorités locales accepteraient au vu de la situation. De telles affirmations sont, bien entendu, difficiles à vérifier, et ce débat dépasse le cadre du présent article. Elles permettent, ceci dit, de comprendre à quel point la question de l’application de la loi est complexe, et d’interroger les multiples intérêts contradictoires qui contribuent à cette complexité. Outre ces questions, il faut noter que, malgré les moments de tension, la question des soins alternatifs n’est pas foncièrement prioritaire, que ce soit au niveau central du système de santé public ou au niveau des organes de décisions locaux : on donne souvent la priorité en termes de temps et de ressources à d’autres questions de santé ou situations plus urgentes. Ceci explique l’ambivalence apparente du système légal mais également, et de façon plus problématique, le vide politique plus général qui entoure la question dans le débat public. De façon générale, il est difficile de distinguer l’acceptation tacite délibérée d’un simple manque d’intérêt pour la question lorsqu’on cherche à expliquer pourquoi les réglementations ne sont pas appliquées – ces deux aspects contribuant probablement à faire perdurer discrètement la situation d’ambiguïté actuelle.

1. **Problématiser la légaliter/l’illégalité**

Tous les participants, même si leurs perspectives divergent, s’accordent sur le fait que le système, en son état actuel, présente d’importantes limites : de facto, une large sphère d’activité s’est développée dans le domaine de la santé, en marge de la légalité et avec très peu de surveillance de la part de l’État. Cependant, si l’on définit les problèmes sur lesquels reposent ces difficultés, les acteurs ont, comme on pouvait s’y attendre, des visions diamétralement opposées et perçoivent le rôle de la science de façon contradictoire.

Ainsi, cette dimension de non-réglementation s’avère hautement problématique pour les autorités publiques, ou les acteurs du biomédical et de la santé publique car les patients ont la possibilité de consulter un praticien en dehors du système biomédical pour répondre à leurs besoins de santé ; lorsque c’est le cas, aucun code, aucune norme ou réglementation commune n’est officiellement imposée aux thérapeutes ; les thérapeutes ne disposent pas non plus d’indications sur la marche à suivre pour établir un lien avec le système de santé principal. Les aspirations de l’État, qui prétend avoir un système de santé fondamentalement articulé autour de pratiques scientifiquement prouvées, sont loin d’être satisfaites. En pratique, des possibilités illimitées s‘offrent aux praticiens, dont les méthodes ne sont pas scientifiquement prouvées, ainsi qu’aux thérapeutes jugés illégitimes, et leur permettent de co-exister parallèlement au système juridique en place peu respecté, dans le contexte de pratiques de santé quotidiennes.

La situation est également jugée insatisfaisante par les thérapeutes alternatifs. Le fait que le droit pénal est rarement appliqué dans des cas de d’exercice illégale de la médecine (ou de la pharmacie) ne rassure pas ceux qui ont constamment à négocier avec cette possibilité. Même si elles sont rares, les poursuites existent et les anecdotes se propagent rapidement. Comme elles sont difficiles à prédire, ces poursuites créent une angoisse chez ceux qui cherchent à exercer leur activité. Bien sûr, même si les poursuites sont rares et les sanctions souvent bien moins importantes que la loi ne le suggère, l’éventualité d’une poursuite crée un sentiment d’insécurité: les études sur la notion de conscience juridique nous rappellent régulièrement que ceux qui sont confrontés à la loi peuvent être intimidés bien au-delà de ce que le système juridique a lui-même anticipé ou de ses conséquences formelles.^[[54]](#footnote-54)^ Les juristes ou les autorités publiques peuvent considérer qu’une poursuite qui entraîne « seulement » un avertissement, ou « seulement » une amende, n’a aucune conséquence. Pour ceux qui sont confrontés à la loi, cet incident peut cependant se révéler beaucoup plus significatif. De la même façon, être officiellement perçu comme un « hors-la-loi » est symboliquement problématique pour de nombreux thérapeutes qui considèrent qu’ils sont de bons citoyens ayant pour but d’exercer dans un cadre de codes de pratiques et de déontologie communs. Pour ces thérapeutes et leurs défenseurs (un petit groupe d’avocats spécialisés qui défendent clairement les mêmes visions), cette précarité et cette illégitimité de pratiques sont souvent présentées comme une atteinte à leur « liberté » et celle de leurs patients (souvent au sens « libertaire »).^[[55]](#footnote-55)^ Dans ce contexte, l’aspect illégitime permet aux thérapeutes de se positionner comme victimes d’un système injuste, ce qui peut également leur être bénéfique en termes de visibilité et de notoriété.^[[56]](#footnote-56)^ Il est ainsi possible que l’illégitimité alimente l’intérêt et soit porteuse d’opportunités.

Enfin, la situation est problématique du point de vue des patients. Un patient recherchant un prestataire de soins alternatifs entre effectivement dans un domaine où les normes sont floues, les directives officielles limitées, outre les rappels d’ordre général de l’État sur les dangers potentiels des thérapies alternatives. Comme seuls les docteurs sont officiellement autorisés à soigner, il n’y pas de recommandation formelle sur ce qui est plus ou moins dangereux, ou au contraire, plus ou moins bénéfique : pour l’État, les lignes établies du biomédical sont les seules qui importent. À ces problèmes bien documentés s’ajoute la question du développement de la légitimité dans le domaine de la santé. Tant que des débats officiels sur les questions réglementaires concernées n’ont pas lieu, le développement de la légitimité (même s’il est fragile) continue de se négocier en marge ou à l’écart des politiques de santé publique, et n’offre pas nécessairement de stratégie cohérente que l’État soit en mesure de contrôler. De plus, l’un des éléments étonnants de la polarisation des débats actuels entre les autorités publiques et les thérapeutes réside dans le fait que la complexité sociale de la relation entre les connaissances, le droit et la médecine est ignorée par les deux parties. De la même façon, les approches pragmatiques qui reconnaissent que le débat n’a pas nécessairement « de solution » (que ce soit grâce au rôle d’arbitre de la science ou grâce à une idée abstraite et détachée de la liberté) mais pourrait faire l’objet d’une médiation, sont visiblement absentes des conversations publiques. Des questions essentielles sont par conséquent laissées à l’écart de la sphère politique : par exemple, l’impact négatif que le manque d’application des lois peut avoir sur la visibilité de certaines pratiques (rendant ainsi les patients plus vénérables) ou le fait que la liberté de choix n’est jamais uniquement une notion autonome et abstraite, mais s’inscrit toujours dans le quotidien et est conditionnée par des (im)possibilités sociales.

La section suivante évoque brièvement certaines des stratégies déployées par les thérapeutes, et a un degré moindre par les entités publiques, pour une réforme de la législation, avant de se pencher sur un facteur clé qui explique les tensions sous-jacentes autour de la légitimité/l’illégitimité des pratiques médicales alternatives : leur positionnement par rapport aux connaissances scientifiques. Puis, l’article conclut qu’une option pour aller de l’avant, dans l’impasse que connaissent les débats actuels, consisterait à ce que les décideurs publics revisitent le rôle de la science dans le développement des stratégies de ce secteur, et les limites de celle-ci comme source de savoir sociétal par rapport à d’autres disciplines (telles les sciences sociales ou humaines).

1. **RÉINVENTER LA LOI : EXIGENCES CONFLICTUELLES ET VIDE POLITIQUE**

Les limites du présent système rendent impératives des discussions pour que celui-ci soit repensé, même si elles émanent de directions radicalement opposées et génèrent de fortes polémiques.

1. **Élargir la loi**

Pour répondre à leur frustration face au système juridique actuel, les thérapeutes se sont organisés pour demander une réforme du droit, développant un ensemble de stratégies pour pouvoir prétendre à la reconnaissance. Pour beaucoup, la route vers la légitimité passe, en définitive, par un changement de position juridique. Premièrement, les nombreux codes de pratiques et les directives officielles que certaines professions ont publiés (par exemple, les herboristes, les naturopathes, les praticiens de médecine chinoise) ont pour objectif d’offrir, non seulement une information, une auto-régulation et des conseils juridiques mais également la facilitation du processus de reconnaissance juridique. Un premier pas nécessaire vers la reconnaissance de l’État consiste à se présenter comme une profession soigneusement auto-réglementée partageant des normes communes, ceci permettant par ailleurs de mettre cette reconnaissance en œuvre plus rapidement. Les professions oeuvrent effectivement en vue d’obtenir ce que Wahlberg appelle « la différenciation de l’intérieur » visant à anticiper un procédé associé à des règlements étatiques du même type dans d’autres domaines. C’est pourquoi, faisant référence au système britannique, Wahlberg nous rappelle que : « Les diverses thérapies MAC ayant été intégrées dans les prestations de santé nationales, on fait de plus en plus appel à leurs praticiens pour aider le public à faire la distinction entre le compétent et l’incompétent dans un cadre pluraliste comptant différentes formes de médecine ».^[[57]](#footnote-57)^

Deuxièmement, les organismes professionnels font pression et recherchent les éventuels soutiens dont ils ont besoin pour être reconnus. Les députés, qui sont ouverts à leurs revendications, contribuent à les réunir au parlement. Les associations de soins travaillent également en collaboration avec les réseaux concernés aux niveaux international et européen (et notamment dans les pays où ils reçoivent davantage d’appuis officiels) pour rechercher de nouveaux soutiens. Ces réseaux internationaux sont particulièrement importants pour les groupes qui considèrent le système français comme un système restrictif sans égal – même s’il est difficile de quantifier l’importance de ces arguments externes sur les rationalités spécifiques de l’État français. Enfin, des moyens de pression indirects sont constamment mis en place : par exemple, au moyen d’inscriptions individuelles de cours de formation ou de professions sur les divers registres des professions (au-delà des professions de santé en France) et par le biais de mesures discrètes, visant à obtenir la reconnaissance professionnelle, qui contournent de manière efficace toute discussion publique plus large.^[[58]](#footnote-58)^

Même si développer ces techniques législatives dépasse le cadre du présent document, il est utile de mentionner quelques caractéristiques. Tout d’abord, s’il existe un degré de coordination entre les associations de thérapeutes alternatifs souhaitant être reconnus, les revendications et les moyens de pression restent fortement le fait de professions individuelles. Les naturopathes, les thérapeutes de médecine chinoise traditionnelle ou encore les herboristes ont, par exemple, créé des alliances distinctes et avancé différentes propositions et requêtes – même s’il y a dans une certaine mesure un recoupement entre le contenu de leurs revendications légales et leurs exigences d’une forme différente de réglementation des professions de santé. Dans le même temps, un petit nombre de juristes professionnels participent à ces discussions avec différentes professions. Leur interprétation reflète les revendications et les techniques décrites ci-dessus, et est en grande partie centrée sur les atteintes du système aux libertés individuelles, mais semble moins disposée, cependant, à s’intéresser de façon explicite à certains problèmes essentiels de ce domaine réglementaire : par exemple, la question de ce qui pourrait constituer des connaissances fiables pour distinguer les praticiens légitimes des praticiens illégitimes, ou les pratiques légitimes et des pratiques illégitimes, ou encore quelles solutions pragmatiques pourraient être déployées pour concilier intérêts de santé publique et croyances individuelles. ^[[59]](#footnote-59)^

1. **Lacunes politiques et limites de l’intervention étatiques**

Face aux activités et au lobbying de haut niveau exercés par les thérapeutes et leurs associations, les stratégies de l’État en matière de médecines alternatives semblent étonnamment limitées. Des moments de friction individuels et des polémiques peuvent survenir, et l’appareil étatique est d’importance dans ce secteur, du moins sur le papier. Malgré une vague d’intérêt de courte durée pour le sujet dans les années 1980,^[[60]](#footnote-60)^ aucun espace ne semble consacré à cette question d’intérêt politique que sont les soins alternatifs. L’accent étant mis, en partie, sur le fait que ces pratiques existent en dehors de la sphère médicale, et des soins officiellement offerts aux patients, elles semblent ne pas relever du domaine de compétence des principales entités de santé publique, et se situent par conséquent entre plusieurs domaines de compétence, à quelques exceptions près. Tout d’abord, la Miviludes a été chargée de mettre en place les directives et les documents-clés relatifs aux soins alternatifs et aux « dérives sectaires ». Or, l’institution manque, d’une part, de personnel face aux enjeux qu’une telle coopération peut représenter (avec uniquement deux conseillers à plein temps chargés des questions de pratiques de santé). D’autre part, il est très clair que le cadre de la mission ne concerne que des cas qualifiés de « dérives sectaires » plutôt que le domaine des soins alternatifs dans son ensemble. Son rôle n’est pas un rôle de santé publique. Deuxièmement, une petite section de la Direction Générale de la Santé est responsable de l’évaluation scientifique des techniques de soins alternatifs : on peut remarquer que le groupe d’appui technique sur les pratiques conventionnelles à visée thérapeutique soit par ailleurs une section du bureau de la qualité des pratiques et recherches biomédicales. Je reviens ci-dessous sur la façon dont cette évaluation scientifique s’inscrit dans la question plus générale de la réglementation des soins alternatifs. Enfin, en 2013, un court rapport sur la place des thérapies en France fut publié par le Centre d’Analyses Stratégiques (CAS), une cellule de réflexion publique en lien avec le bureau du Premier Ministre. Le rôle du CAS est de soumettre des rapports sur diverses questions d’affaires courantes pouvant attirer l’attention des pouvoirs publics. Il s’agit de non-specialistes travaillant sur toute une série de questions, parmi lesquelles seul un petit nombre de propositions est retenu par le gouvernement pour des recherches ou des discussions politiques plus poussées. En ce sens, le rapport produit sur les thérapies alternatives en est un parmi d’autres et non pas le résultat d’une action à plus long terme ou d’une stratégie politique. Toutefois, ce document reste unique dans ses propositions pour repenser les stratégies actuelles ainsi qu’une première étape vers une réflexion collective.^[[61]](#footnote-61)^ A ce jour, les autorités sanitaires ne se sont pourtant pas saisies du rapport et il ne constitue rien de plus qu’un ensemble de propositions. Il fut, cependant, examiné en profondeur par la Commission du Sénat mentionnée ci-dessus, rappelant de façon brutale la nature polémique de ces débats. Malgré un rapport ne suggérant guère plus qu’une réflexion dans un domaine politique où le système juridique ne représente tout simplement pas les expériences sociétales, la Commission a critiqué les auteurs de façon véhémente pour la “dangerosité” de leurs propositions.^[[62]](#footnote-62)^

Dans la section ci-dessous, je soutiens que l’absence relative d’engagement de l’État face aux questions en jeu est également symptomatique du fait que ces questions politiques sont réduites à des problèmes de « savoir scientifique objectif ». La science peut, certes, fournir un certain nombre de précieux indices pour délimiter les frontières de la légitimité en matière de santé, mais elle ne suffit pas pour répondre aux défis de société soulevés par la question des pratiques alternatives. Dans un domaine aussi fortement façonné par des influences socio-culturelles et historiques, il est essentiel que les États soient prêts à s’engager sur la question difficile de la légitimité sociale en matière de santé, et de repositionner le rôle de la science dans ce cadre – voire ses éventuelles limites.

1. **Politiques du Savoir et comment négotier avec la science**

Cette partie se penche sur la question fondamentale de la « place » de la science dans la réglementation des soins alternatifs en France. Elle affirme que se baser essentiellement sur la science comme moyen d’arbitrage pour régler les dilemmes en matière de législation, a entraîné une action politique insuffisante. La biomédecine constitue, pour le moment, la source essentielle de savoir utilisée pour délimiter les frontières entre les soins légitimes et illégitimes. Cette dépendance à l’égard de la science est particulièrement marquée dans les discours publics français, et explique certaines de ces particularités. Par exemple, les débats au sein du Sénat traduisent une peur profonde qui consiste à suggérer que l’idéal républicain d’un État rationnel pourrait être fondamentalement menacé si les professionnels du biomédical n’étaient plus les seuls prestataires de soins :

« Les 18^ème^ et 19^ème^ siècles ont vu la transition entre « l’Hotel-Dieu » et l’hôpital public. L’héritage des Lumières a permis l’essor de la médecine scientifique occidentale. Le 21ème siècle risque de générer le glissement de « l’Hôpital public » vers « l’autel des gourous ». Les Lumières seraient alors éteintes par l’obscurantisme sectaire que les méthodes thérapeutiques illusoires contribuent à diffuser auprès d’un public avide et de plus en plus influençable !”^[[63]](#footnote-63)^

"Le relativisme rend donc les superstitions aussi valides que les sciences. La science va même être reléguée au rang de mythe et les critères de rationalité seront désormais présentés comme des contingences d’une culture relative. On tombera alors bien bas !”^[[64]](#footnote-64)^

"Depuis le début du dernier tiers du 20ème siècle et l’expérience des totalitarismes, on assiste à une remise en cause de la légitimité du fondement scientifique comme si devait être oubliée l’exigence de l’« essai thérapeutique » reposant sur l’emploi du placebo et l’étude dite « prospective, randomisée et en double aveugle ». Les postulats vont ici remplacer les preuves. »^[[65]](#footnote-65)^

Les ideaux républicains sont transcrits ici dans le contexte particulier de la santé. À leur tour, les réponses étatiques à la question de la médecine alternative visent, de la même façon, à établir la validité scientifique des thérapies alternatives, en utilisant les tests scientifiques de la biomédecine – ce procédé n’est pas inhabituel en soi, mais ce qui est propre à la France, c’est l’absence d’autres sources claires d’inspiration dans le domaine de la réglementation. On présente la science comme la meilleure façon, voire la seule, de réguler les limites entre les prestations de santé légitimes et illégitimes. Toutefois, ceci risque de ne pas être suffisant, s’il on aborde la question non seulement d’un point de vue scientifique mais également sous un angle social.

La question de la validité scientifique est, bien évidemment, capitale dans le domaine de la santé. Parallèlement, l’emploi de médecines alternatives n’est pas uniquement lié à l’aspect scientifique mais probablement motivé par un rejet social du regard clinique. Dans une certaine mesure, il n’est pas possible de répondre au besoin d’altérité exprimé par les patients uniquement par le biais de la science. Ceci s’exlique a 2 niveaux : premièrement, l’ensemble des essais et des justifications que déploie la biomédecine risque de ne pas suffire pour juger des modes alternatifs de connaissance. Deuxièmement, lorsque le choix du patient est différent de celui de l’État, ou des propositions de la science, il semble nécessaire de s’interroger sur la gestion possible de ces choix dans le cadre d’objectifs de santé publique, plutôt que de chercher uniquement à les interdire ou à les combattre. En d’autres termes, même si l’on est sceptique quant aux médecines alternatives, la question de la réglementation devra assurément s’appuyer sur d’autres savoirs que ceux de la science et de la biomédecine, tels que les connaissances en sciences sociales. Jusqu'à présent, l’État français a centré son approche stratégique sur l’interdiction, du moins théorique, pour ceux qui opèrent en dehors du cadre de toute preuve scientifique. De larges espaces de pratique thérapeutique ont, par conséquent, échappé à toute réglementation ou intervention étatique. De même, l’État ne propose aucune hiérarchisation ou aucun tri entre les diverses pratiques non-biomédicales, ou entre les différentes professions, et il n’existe aucune position officielle concernant l’utilité pathologique ou, au contraire, la dangerosité ou le manque de fiabilité de certaines pratiques. Aux yeux de l’État, les seuls critères pertinents pour définir les frontières officielles de la légitimité reposent sur la formation biomédicale reçue ou sur des éléments d’ordre « scientifique » ou « non-scientifique ». Malgré leur relation ambivalente face à la science, les thérapeutes alternatifs ont eux-mêmes placé la science au centre de la plupart de leurs discours. D’un côté, ils insistent sur les lacunes de la biomédecine, et proposent quelque chose de différent à travers une autre conception du monde. Dans le même temps, ils entendent démontrer la validité de leurs revendications de façon à intégrer le système médical principal, en utilisant les modalités du dit système. Le langage scientifique est utilisé à ces fins et la mise en place de preuves scientifiques, dans le but de soutenir leurs revendications, constitue un élément central de la stratégie en matière de légitimation.^[[66]](#footnote-66)^

Inévitablement, il existe des façons multiples de trouver un équilibre au sein des diverses professions, certains sous-groupes de thérapeutes se concentrant davantage sur leur différence et l’aspect traditionnel de leur pratique quand d’autres insistent sur la scientificité.^[[67]](#footnote-67)^ L’enjeu est de maintenir un équilibre judicieux entre la crédibilité aux yeux des régulateurs potentiels, et l’abandon de la différence, comme ce fut le cas dans d’autres contextes. Lyioha nous rappelle ainsi que « la reconnaissance statutaire de l’ostéopathie et de la chiropraxie au Royaume-Uni a imposé le rejet des fondements ésotériques de ces thérapies ». Lorsque les identités scientifiques et alternatives sont en jeu, les thérapeutes se doivent de décider à quel point ils acceptent la transformation pour obtenir la réglementation, et comment les ajustements et les identités peuvent être négociés.

L’actuelle formulation des questions en jeu – notamment de la part de l’État qui insiste sur la question de la preuve scientifique - ignore les aspects sociaux qui mènent les patients à des épistemologies alternatives de soins. De même, établir la réglementation ou l’utilisation de techniques différentes, parallèlement à celles dont l’efficacité scientifique est prouvée, requiert un ensemble de compétences et de connaissances telles que celles proposées par les spécialistes des sciences sociales et les anthropologues qui se sont, au fil des années, penchés sur la question des thérapies non-biomédicales. En d’autre terme, la science peut certes répondre à la question de la valeur scientifique prouvée de telle ou telle pratique. Elle ne peut pas répondre à la question plus pragmatique de la meilleure réglementation possible pour des pratiques non prouvées que des patients continuent, malgré tout, et sans attente ‘scientifique’, à utiliser. Ce sujet doit, en conséquence, atteindre les sphères politiques adéquates et être abordé comme un problème de société qui requiert l’attention urgente de l’État, toutes connaissances et disciplines confondues. Pour l’heure, il semble que l’État français continue de s’appuyer sur la biomédecine afin de définir ses propres limites d’acceptabilité, et ce en craignant que règlementer le ‘non-scientifique’ de manière plus créative, explicite et peut-être pragmatique ne court le risque de mettre la science en tant qu’institution en péril.

Ceci permet, par conséquent, aux défenseurs les plus virulents des traitements alternatifs de générer les propositions actuelles de réinvention du système juridique. Une approche plus pragmatique, qui correspondrait par ailleurs à la démarche de l’État de développer un savoir et des solutions non partisans, consisterait à faciliter une action pragmatique et pluridisciplinaire, non seulement par le biais du savoir scientifique en tant que tel, mais aussi grâce aux connaissances des sciences sociales particulièrement bien placées pour appréhender la question de la diversité dans le domaine médical comme phénomène de société.

1. CONCLUSION

Le cadre juridique qui régit actuellement les thérapies alternatives en France est truffé d’ambivalence. Il existe une limite formelle et stricte entre la possibilité donnée aux professionnels, dont la formation biomédicale permet d’offrir un ensemble de thérapies (y compris certaines pratiques complémentaires et alternatives), et l’interdiction pour pratiquement tous les autres de traiter ou de diagnostiquer les patients. La réalité quotidienne dans le domaine des soins est, cependant, en contradiction avec ces données juridiques : parallèlement aux médecins acupuncteurs, homéopathes ou autres dont les pratiques sont légalement acceptées, les patients s’adressent à bon nombre de praticiens qui ne remplissent pas les conditions requises par la loi. Ce faisant, alors qu’ils se trouvent officiellement en marge d’un système légal basé sur le monopole et l’interdiction générale, il n’existe guère d’indications relatives à ce qui est plus ou moins fiable, ou plus ou moins dangereux, et ceci est valable pour les pratiques comme pour les praticiens. Selon l’État, ceux dont la pratique n’est pas suffisamment avérée sur le plan scientifique, ne sont pas aptes à prodiguer les soins qu’ils cherchent à promouvoir. Au cours des années, les thérapeutes ont appris à contourner cette illégalité et ont développé, à la fois individuellement et collectivement, des outils pour préserver le consensus d’ambivalence qui rend certaines pratiques possibles, et répandues, malgré l’illégalité de leur statut officiel. Ce système est une source de frustration pour d’autres qui le considèrent risqué pour les patients évoluant dans cet espace de soins non réglementé, en marge du système biomédical, et qui soulignent la nécessité de mieux appliquer les règles. Cependant, à l’heure actuelle, aucun espace politique qui permettrait d’explorer des solutions réglementaires ne semble avoir été véritablement défini, probablement du fait même que les pratiques alternatives ne constituent pas une ressource acteulle du système de santé officiel. Par ailleurs, les échanges entre deux positionnements fortement polarisés restent polémiques, l’un défendant la notion abstraite de « liberté de choix », l’autre l’aspect central que joue la science dans le domaine des prestations de soins ainsi que les dangers susceptibles d’entamer cette centralité. Jusqu’à présent, il y a, en France, très peu d’engagement pragmatique par rapport à la crise réglementaire qui touche le secteur de la santé. Un tel engagement est absolument nécessaire, même s’il requiert également des ressources dont la science seule ne dispose pas.^[[68]](#footnote-68)^ Il est difficile de résoudre cette crise juridique sur la question des limites des traitements médicaux, notamment parce que le secteur de la santé doit, dans un premier temps, être repensé en tant qu’espace social, avant que la relation entre le droit et la médecine puisse être définie grâce à des outils autres que ceux de la science. Ici, l’enjeu pour les juristes n’est pas d’ordre médical ou scientifique, les solutions ne sont pas non plus déterminées par des critères scientifiques et ne représentent pas nécessairement une menace pour le consensus scientifique. En revanche, la question est d’intégrer des pratiques qui ne sont pas prouvées scientifiquement mais que les patients choisissent, cependant, d’adopter, et de concevoir un système réglementaire qui prend cette situation en compte sans nécessairement mettre en péril l’importance du savoir biomédical actuellement au cœur des prestations de soins. Au centre de ces discussions se trouve la question de la modernité de la médecine et comment cette dernière peut être envisagée par la loi : des visions divergentes entre ceux qui cherchent à maintenir une vision de modernité à travers des critères scientifiques, et ceux qui considèrent la modernité comme multidirectionnelle et souhaitent tenir compte de conceptions alternatives. Le défi pour l’État contemporain consiste à se pencher sur la façon de satisfaire diverses conceptions de la modernité dont un aspect contemporain est le retour aux traditions, à la nature ainsi qu’un certain questionnement de l’institution scientifique. Quant aux lois dans le domaine médical, le défi consiste à produire un système réglementaire qui tienne compte du fait que les tensions sociétales s’opposent aux hypothèses téléologiques de la science. Alors que certains peuvent ne pas accepter la remise en cause des connaissances scientifiques, apprendre à négocier malgré ces obstacles et établir de nouvelles lignes de légitimité conditionnée pourrait s’avérer inévitable.^[[69]](#footnote-69)^ Bon nombre d’options pragmatiques et impartiales peuvent être élaborées, à condition à la foisd’exploiter les connaissances dont disposent les sciences sociales en matière de pratiques de soins alternatives et leurs complexités sociales, et de mettre en place un espace politique bien défini pour les explorer. Il est bien évidemment impossible de donner forme à ces recommandations ou de développer des propositions concrètes dans ce document, de même qu’il n’existe pas de stratégie miracle. Quelques suggestions peuvent toutefois être formulées, au travers de quatre points clés. Premièrement, et comme ceci est suggéré ci-dessus, un débat interdisciplinaire s’avère nécessaire pour etudier la question de la réglementation des médecines alternatives. Il est essentiel de s’appuyer sur un éventail d’expertises pour élargir le débat et ne pas en rester uniquement à des questions de preuve scientifique. Idéalement, il faudrait que les autorités de santé publiques (sous l’auspice du ministère de la santé par exemple) organisent le débat et invitent des chercheurs ayant les compétences nécessaires à y participer pour traiter la question réglementaire dans toute sa complexité ainsi que la dimension sociétale de la santé. Deuxièmement, la première mission d’une telle cellule d’action devrait consister à « ouvrir la boîte des connaissances » sur le sujet donné : comme le présent document a tenté de le démontrer, les ramifications actuelles du système juridique dans son aspect pratique, sont extrêmement complexes et il reste beaucoup à apprendre (y compris grâce au cours du projet particulier sur lequel repose cet article). Néanmoins, en tenant compte de ce que nous avons déjà appris des thérapies alternatives et des débats passionnés qui ressortent occasionnellement en France, il est clair qu’il s’agit d’un problème de société, et de santé publique, qui se doit d’être considéré comme tel et pris au sérieux. Alors que les discussions interdisciplinaires sur le sujet progressent, il est primordial d’intégrer la recherche dont nous disposons dans le domaine des sciences sociales, ou de commanditer de nouvelles recherches, et de les inclure dans le débat qui a lieu avec les autorités de santé publique. La création du groupe d’appui technique sur les pratiques non-conventionnelles à visée thérapeutique, mentionné précédemment, a ouvert la porte à de nouvelles recherches dans ce domaine, les réponses recherchées se limitant néanmoins aux questions de la preuve et de l’efficacité scientifique. Ceci ne peut constituer, , comme on l’a rappellé ici à plusieurs reprises, qu’un aspect des enjeux. Les sciences sociales pourraient contribuer à cette recherche et les résultats d’études préalables pourraient être intégrés au dialogue. Troisièmement, la question de la preuve est précisément celle qui requiert une attention particulière durant ces discussions. Comme il est indiqué ici ou démontré par d’autres, les moyens scientifiques irréfutables et la preuve de l’efficacité ne suffisent pas toujours à résoudre les questions de légitimité, voire même d’utilité, en matière de santé. Les stratégies axées sur les patients invoquent, par exemple, des méthodes qualitatives alors que l’expérience du patient repose sur un ensemble d’hypothèses concernant l’efficience (plutôt que l’efficacité en soi). On pourrait également reconsidérer la question de l’effet placebo en tant que forme particulière d’efficacité sociale et pas uniquement comme élément dépourvu de base scientifique. De nouvelles décisions en matière de remboursement ou de non-remboursement ne seraient pas à pré-juger pour autant, mais pourraient faire partie d’une nouvelle réflexion autour des effets du système de santé (en tant qu’il engloberait non seulement la guérison en tant que telle, mais également une vision plus globale du ‘soin’). Cela ne signifie pas non plus que tous les systèmes de preuve ont inévitablement une valeur égale (et qu’il conviendrait donc comme certaines citations ci-dessus le suggèrent, d’abandonner toute « rationalité scientifique »). Au contraire, cela signifie que différentes formes de connaissances complémentaires pourraient avoir différents usages. Le quatrième et dernier point concerne la question des techniques juridiques et réglementaires auxquelles il serait bon de porter une attention particulière lors de ces discussions. L’enjeu fondamental consiste ici à réinventer les systèmes de réglementation dans le but de proposer un système réglementaire durable qui prenne en compte l’aspect social des pratiques. Il est impératif d’inclure aux débats les connaissances socio-juridiques dont le caractère est essentiel. L’une des difficultés qui accompagnera l’ouverture du système résidera dans le fait qu’il faut décider où et comment délimiter les « lignes rouges ». En effet, lorsqu’on s’appuie essentiellement sur des méthodes scientifiques et des tests d’efficacité, il est relativement facile de définir les lignes de l’acceptabilité ou de déterminer ce qui est « réél » ou « faux ». Si l’on décide d’inclure de nouvelles formes de preuve dans le système juridique qui entoure la médecine, et d’accroître le rôle des connaissances sociologiques et des méthodes qualitatives dans les stratégies réglementaires, définir les limites de la légitimité n’en sera inévitablement que plus compliqué. Le système réglementaire devrait ainsi définir des lignes plus nuancées afin de déterminer le rôle de telle personne ou de telle pratique et de spécifier les conditions dans lesquelles peut se développer cette vision élargie du système de santé. Certaines questions devront être repensées, telles que celles définissant les critères de légitimité de professions émergentes ou encore comment inclure ces dernières aux structures institutionnelles ; comment déterminer la portée des revendications il/légitimes si l’on dépasse la vision étriquée de l’efficacité scientifique ; qui est en droit d’utiliser certains titres ou de revendiquer certaines compétences ; comment définir des systèmes conçus sur mesure pour le suivi et l’application (et comment définir les rôles respectifs des professions et de l’État)  de nouvelles normes; comment définir de nouvelles formes de réglementation en termes de publicité et d’informations relatives aux patients etc… Ces questions invitent à prêter une attention toute particulière aux techniques réglementaires, à aller au-delà des limites légales/illégales qui paraissent difficiles à maintenir et continuent de créer un climat d’incertitude quotidien. Le projet sur lequel repose ce document continuera d’explorer ces questions, en incluant au dialogue les diverses façons dont des dilemmes comparables ont été traités, à la fois, en France et dans chacune des autres études de cas.

1. Professeure Émilie Cloatre, Faculté de Droit de l’Université du Kent, Canterbury CT1 2PZ Ryaume-Uni, [e.cloatre@kent.ac.uk](mailto:e.cloatre@kent.ac.uk) [↑](#footnote-ref-1)
2. Mon analyse est influencée ici par des conceptions STS (Science, Technologie et Société) en matière de developpement de la légitimité de la pratique scientifique, par exemple M. Callon, P. Lascoumes and Y. Barthe, Agir dans un monde incertain: Essai sur la Démocratie Technique (Massachusetts Institute of Technology 2001) ou S. Jasanoff, The Fifth Branch: Science Advisers as Policy-Makers, Harvard University Prèss, 1998 [↑](#footnote-ref-2)
3. A. Wahlberg, 'A Quackery with a Difference-New Médical Pluralism and the Problem of ‘dangerous Practitioners’ in the United Kingdom', (2007) 65 Social Science and Medicine 2307-2316 [↑](#footnote-ref-3)
4. ^3^ M. Ramsey ‘Alternative Medicine in Modern France’ (1999) 43 Médical History 286-322, p.320 [↑](#footnote-ref-4)
5. Voir par exemple: J. McHale, 'Legal frameworks, professional regulation and CAM practice in England and Wales: is CAM ‘the special one’?', in N. Gale & J. McHale (ed.), Routledge Handbook of Complementary and Alternative Medicine: Perspectives from social science and law (2015), [↑](#footnote-ref-5)
6. ^5^ Pour toute discussion sur l’histoire et les contextes de telles terminologies, consulter M. Ramsey (1999) [↑](#footnote-ref-6)
7. Bourse Wellcome 200380/Z/15/Z [↑](#footnote-ref-7)
8. S.A. Cole & A. Bertenthal, 'Science, Technology, Society, and Law', (2017) 13 Annual Review of Law and Social Science 351–371; E. Cloatre & M. Pickersgill (ed.), Knowledge, Technology and Law (2014)  [↑](#footnote-ref-8)
9. N. De Genova, 'Spectacles of Migrant ‘Illégality’: The Scene of Exclusion, the Obscene of Inclusion', (2013) 36 Ethnic and Racial Studies 1180–1198 >; D. Cooper, 'Institutional Illégality and Disobedience: Local Government Narratives', (1996) 16 Oxford Journal of Legal Studies 255–274; E. Cloatre and M. Enright “On the Perimétér of [↑](#footnote-ref-9)
10. Par exemple: Ayo Wahlberg (2007); McHale (2015); N. Gale, 'The Sociology of Traditional, Complementary and Alternative Medicine: Traditional, Complementary and Alternative Medicine', (2014) 8 Sociology Compass 805– 822 [↑](#footnote-ref-10)
11. Wahlberg , 2007  [↑](#footnote-ref-11)
12. P. Cohen & I. Rossi, 'Le pluralisme thérapeutique en mouvement: Introduction du numéro thématique

    « Anthropologie des soins non-conventionnels du cancer »', (2011) 2 Anthropologie et Santé (online)  [↑](#footnote-ref-12)
13. P. Elzière, 'Des médecines dites naturelles', (1986) 4 Sciences sociales et santé 39–74 [↑](#footnote-ref-13)
14. A. Grisoni, 'De la naturopathie rurale à la santé naturelle: distanciation et assimilation autour de la notion d’espace', (2012) 8 Nouvelles perspectives en sciences sociales 237; A. Grisoni, 'Sous les pavés, la terre : culte du bien-être et nouveaux métiers : la naturopathie en transformation à la conquête du marché. Doctorat de sociologie, École des hautes études en sciences sociales (EHESS)(2012); A. Marcellini et al, 'Itinéraires thérapeutiques dans la société contemporaine. Le recours aux thérapies alternatives: une éducation à un « autre corps »?’ (2000) 5 Corps et culture 1-15 > [↑](#footnote-ref-14)
15. A. Marcellini et al, (2000) ; Gentis R. (1980) Leçons du corps, Paris, Flammarion; Van der Gest and Reynolds Whyte (2003)  [↑](#footnote-ref-15)
16. Eg. See the approach taken in 1986 by the Ministry of Social Affairs and Solidarity’s Groupe de Réflections sur les ‘Médecines différentes’. For UK conversations on these issues see for example the House of Lords Science and Technology Select Committee Report (2000) Sixth Report: Complementary and Alternative Medicine, HL: 123; Complementary and Natural Healthcare Council (2013) The First Five Years, London: The Complementary and Natural Healthcare Council. [↑](#footnote-ref-16)
17. P. Pinell, 'Modern Medicine and the Civilising Process.', (1996) 18 Sociology of Health and Illness 1–16 [↑](#footnote-ref-17)
18. J. Léonard, 'La médicalisation de l’État : l’exemple des premières décennies de la IIIe République', (1979) 86 Annales de Bretagne et des pays de l’Ouest 313–320.  [↑](#footnote-ref-18)
19. “Commission d’enquête sur l’influence des mouvements à caractère sectaire dans le domaine de la santé”. [↑](#footnote-ref-19)
20. Sénat (2013) Rapport Fait au Nom de la Commission d’Enquête sur l’Influence des Mouvements à Charactère Sectaire dans le Domaine de la Santé, Tome 2: Procès Verbaux des Auditions’, Session Ordinaire 2012-2013 N.480 [↑](#footnote-ref-20)
21. Article L. L4161-1 du Code de la Santé Publique. For comments see for example: Ramsey (1999); B. Lavaud- Legendre, 'Charlatanisme et droit pénal', (2008) Les Tribunes de la santé 67–75 [↑](#footnote-ref-21)
22. Article L 4161-5 du code de la santé publique [↑](#footnote-ref-22)
23. For examples of relevant case-law, and the broad définition of the offence being applied: Cour de cassation, Chambre criminelle, 28 juin 2016, 15-83.587; Cour de cassation, Chambre criminelle, 9 mars 2010, 09-81.778; Cour de cassation, Chambre civile 1, 16 octobre 2008, 07-17.789; Cour de Cassation, Chambre criminelle, du 21 septembre 2004, 04-80.526; Cour de Cassation, Chambre criminelle, du 2 juin 2004, 03-87.815 [↑](#footnote-ref-23)
24. Cambrella, Légal Status and Regulation of CAM in Europe, Part 1: CAM Regulations in the European Countries (2012), available at: https://phaidra.univie.ac.at/view/o:291583 [↑](#footnote-ref-24)
25. For a historical review of the pharmaceutical monopoly, see: M.D. Campion, 'Les résonances actuelles de la loi de Germinal. Monopole pharmaceutique et exercice illégal de la pharmacie', (2003) 91 Revue d’histoire de la pharmacie 395–406 [↑](#footnote-ref-25)
26. Article L. 4211-1 Code de Santé Publique [↑](#footnote-ref-26)
27. Décret 2008-841 [↑](#footnote-ref-27)
28. Cour de cassation, criminelle, Chambre criminelle, 20 septembre 2011, 10-83.649; Cour de cassation, criminelle, Chambre criminelle, 22 février 2011, 10-81.359. See also Bureau, 2015 [↑](#footnote-ref-28)
29. “La Miviludes observe et analyse le phénomène sectaire, coordonne l’action préventive et répressive des pouvoirs publics à l’encontre des dérives sectaires, et informe le public sur les risques et les dangers auxquels il est exposé.” Miviludes website, homepage: http://www.derives-sectes.gouv.fr [↑](#footnote-ref-29)
30. The spiritual or religious origins of some specific practices, such as naturopathie, have made them particular points of attention of the Miviludes and associated policies – see for example: Grisoni, "De la naturopathie rurale à la santé naturelle" (2012)  [↑](#footnote-ref-30)
31. J.P. Chantin, 'Les sectes en France. Marges et dissidences', (2000) 66 Vingtième Siècle. Revue d’histoire 67– 78; F. Champion & M. Cohen, 'Les sociologues et le problème des dites sectes / Sociologists and the ‘So-called’ Sect Issue', (1996) 96 Archives des sciences sociales des religions 5–15 [↑](#footnote-ref-31)
32. Miviludes (2012) Santé et Dérives Sectaires, Paris: Miviludes (available at http://www.derives- sectes.gouv.fr/missions/actualites/guide-santé-et-dérives-sectaires ) [↑](#footnote-ref-32)
33. Loi n.2002-303 du 4 Mars 2002 relative aux droits des malades et à la qualité du système de santé 11 [↑](#footnote-ref-33)
34. M. Saks, 'Power and Professionalisation in CAM: a sociological approach', in J. McHale & N. Gale (ed.), Routledge Handbook of Complementary and Alternative Medicines: perspectives from social science and law (2015), 30 [↑](#footnote-ref-34)
35. Pour des exemples de trajectoires menant les médecins à une formation en acupuncture, consulter: F. Parent, « Seuls les médecins se piquent d’acupuncture » ?', (2015) N° 25 Terrains & travaux 21–38 Sur l’homéopathie, consulter par exemple Lazarus 2007 [↑](#footnote-ref-35)
36. O. Faure, 'Une histoire de l’homéopathie', (1990) 27 Vingtième Siècle, Revue d’histoire 116–117 [↑](#footnote-ref-36)
37. Ramsey (1999)  [↑](#footnote-ref-37)
38. See for example Fagon J-Y and Viens-Bitker C, 2012, “Médecines Complémentaires à l’Assistance Publique- Hôpitaux de Paris : Rapport du Comité ‘d’Orientation”, available at http://cme.aphp.fr/sites/default/files/CMEDoc/cme-10-07-2012_medecines_complementaires.pdf (last accessed 23rd March 2017) [↑](#footnote-ref-38)
39. Tribune, 18th March 2018, « L’appel de 124 Professionnels de la Santé cone les “Médecines Alternatives” » available at : http://www.lefigaro.fr/vox/societe/2018/03/18/31003-20180318ARTFIG00183-l-appel-de-124-professionnels-de-la-sante-contre-les-medecines-alternatives.php. The Conseil National de l’Ordre des Médecins responsded on the 22nd March : https://www.conseil-national.medecin.fr/node/2689 [↑](#footnote-ref-39)
40. Sur les questions de différence épistémologique, consulter par exemple: V. Adams, 'Randomized Controlled Crime: Postcolonial Sciences in Alternative Medicine Research', (2002) 32 Social Studies of Science 659–690 [↑](#footnote-ref-40)
41. C.R. Janes, 'The Health Transition, Global Modernity and the Crisis of Traditional Medicine: The Tibetan Case', (1999) 48 Social Science & Medicine 1803–1820 [↑](#footnote-ref-41)
42. Les tensions initiales sur le rôle des médecins dans l’utilisation de techniques non-biomédicales figurent également dans les travaux de recherche consacrés aux débuts de l’acupuncture en France (consulter par exemple L. Candelise ‘Construction, acculturation etdiffusion de l’« acupuncture traditionaliste française » au XXe siècle’ (2008) 16 Documents Pour l’Histoire des Techniques (online)). [↑](#footnote-ref-42)
43. Borrowing here from legal consciousness vocabulary and its emphasis on the multiple expressions of law in everyday experiences (see for ex. Silbey and P. Ewick The Common Place of Law, Chicago: University of Chicago Press (1998))  [↑](#footnote-ref-43)
44. Ramsey (1999) [↑](#footnote-ref-44)
45. Marcellini et al. (2000)  [↑](#footnote-ref-45)
46. Certaines organisations professionnelles sont critiques à l’égard de telles approches, et considèrent qu’elles les rendent moins crédibles en tant que professionnels de la santé, dans un pays où les médecins n’ont pas le droit de faire la promotion de leurs propres services [↑](#footnote-ref-46)
47. Sénat (2013) pp.175-185 [↑](#footnote-ref-47)
48. Exemple: Cour de cassation, Chambre criminelle, 16 décembre 2014, 14-80.088; Cour de cassation, Chambre criminelle, 9 février 2010, 09-80.681 [↑](#footnote-ref-48)
49. E. Cloatre and M. Enright ‘On the Perimeter of the Lawful: Enduring Illegality and the Irish Family Planning Movement 1972-1985’, Journal of Law and Society 44(4) pp. 471-500 [↑](#footnote-ref-49)
50. A.C. Hoyez, '« L’ayurveda, c’est pour les Français ». Interroger recours aux soins, systèmes de santé et expérience migratoire', (2012) 28 Revue européenne des migrations internationales 149–170 [↑](#footnote-ref-50)
51. Parent (2015) [↑](#footnote-ref-51)
52. Ibid [↑](#footnote-ref-52)
53. Grisoni (2012) [↑](#footnote-ref-53)
54. S. Silbey and P. Ewick (1998) [↑](#footnote-ref-54)
55. A. Lazarus & G. Delahaye, 'Médecines complémentaires et alternatives: une concurrence à l’assaut de la médecine de preuves?', Les Tribunes de la santé 79–94 [↑](#footnote-ref-55)
56. Ramsey (1999) [↑](#footnote-ref-56)
57. Wahlberg (2007)  [↑](#footnote-ref-57)
58. Par exemple, une étape administrative importante pour les écoles et les centres de formation consiste à obtenir la reconnaissance de leur programme par le Répertoire National des Certifications Professionnelles (RNCP). Cette certification, obtenue grâce à un système mis en place par le Ministère du Travail (ie Ministry of Works and Pensions), n’a aucun rapport avec le Ministère de la Santé, ou une quelconque reconnaissance de la valeur d’une thérapie. La reconnaissance du RNCP est souvent, néanmoins, présentée par les associations de soins alternatifs comme un élément d’importance dans la reconnaissance des ‘professions’. Le rôle précis que certaines procédures jouent dans l’élaboration de la légitimité devient flou à mesure que leurs technicalités s’effacent au profit d’une référence plus large liée aux processus de ‘reconnaissance de l’État’ [↑](#footnote-ref-58)
59. Par exemple I. Robard, Médecines non-conventionnelles et droit, Litec, 2002 [↑](#footnote-ref-59)
60. Elzière (1986) [↑](#footnote-ref-60)
61. Centre d’Analyse Strategique, (2012) ‘Quelle réponse des pouvoirs publics à l’engouement pour les médecines non-conventionnelles?’, Note d’Analyse 290 available at: http://archives.strategie.gouv.fr/cas/system/files/2012-10-02-_medecinesnonconvetionnelles-na290_0.pdf Verifier autres initiatives Ibid [↑](#footnote-ref-61)
62. Sénat, 2013,pp.206-2016 [↑](#footnote-ref-62)
63. Sénat (2013) p.75 [↑](#footnote-ref-63)
64. Sénat (2013), p. 67 [↑](#footnote-ref-64)
65. Sénat (2013), p. 70 [↑](#footnote-ref-65)
66. Grisoni (2012) [↑](#footnote-ref-66)
67. Parent (2015) [↑](#footnote-ref-67)
68. Cohen & Rossi, Le pluralisme thérapeutique en mouvement (2011) [↑](#footnote-ref-68)
69. Guilloux, Grisoni [↑](#footnote-ref-69)
